# Phytoplankton dynamics in a shellfish farming lagoon in a deltaic system threatened by ongoing climate change

**DOI:** 10.1038/s41598-024-70492-6

**Published:** 2024-08-21

**Authors:** Francesco Bolinesi, Emanuele Rossetti, Olga Mangoni

**Affiliations:** 1 Dipartimento di Biologia, Università degli Studi di Napoli Federico II, Complesso di Monte Sant’Angelo, Via Cinthia 21, 80126 Napoli, Italy; 2 CoNISMa, Piazzale Flaminio 9, 00196 Roma, Italy; 3 Consorzio Cooperative Pescatori del Polesine O.P. S.C.Ar.L., 45018 Scardovari, Rovigo, Italy

**Keywords:** Chemotaxonomy, Functional traits, Po Delta, Biodiversity, Monitoring of coastal marine systems, Ecology, Ecology, Environmental sciences, Ocean sciences

## Abstract

Global climate change has generated an increasing number of environmental problems, especially in Mediterranean coastal areas, such as the Po Delta (PD), where shellfish production has undergone an overall decline because of strong environmental changes. The present study is centred on assessing the fundamental ecological aspects in one of the most crucial European shellfish production lagoons, Sacca degli Scardovari (SC), addressing phytoplankton community parameters directly affecting shellfish production, namely, chemotaxonomic composition, size fractions, and total biomass, in relation to the physicochemical properties of the water column and mussel filtering activity. Our findings suggest that the phytoplankton community structure, its role within the lagoon food web and its production cycles depend on two distinct allogenic inputs, which shape the community differently and exert substantial control on shellfish production. At the same time, the suspended mussel biomass strongly controls the phytoplankton size composition, as their growth is largely supported by nanophytoplankton. As the Po River collects the drainage waters of the Italian side of the entire Alpine Arch, the phytoplankton dynamics reported here represent a useful baseline for further addressing issues of climatic changes affecting lagoon ecology. We believe that our study presents an innovative tool for the planning and management of interventions aimed at enhancing national mussel production without neglecting aspects of environmental protection or the integrity of the coastal system, with significant scientific implications.

## Introduction

**Fig. 1:**
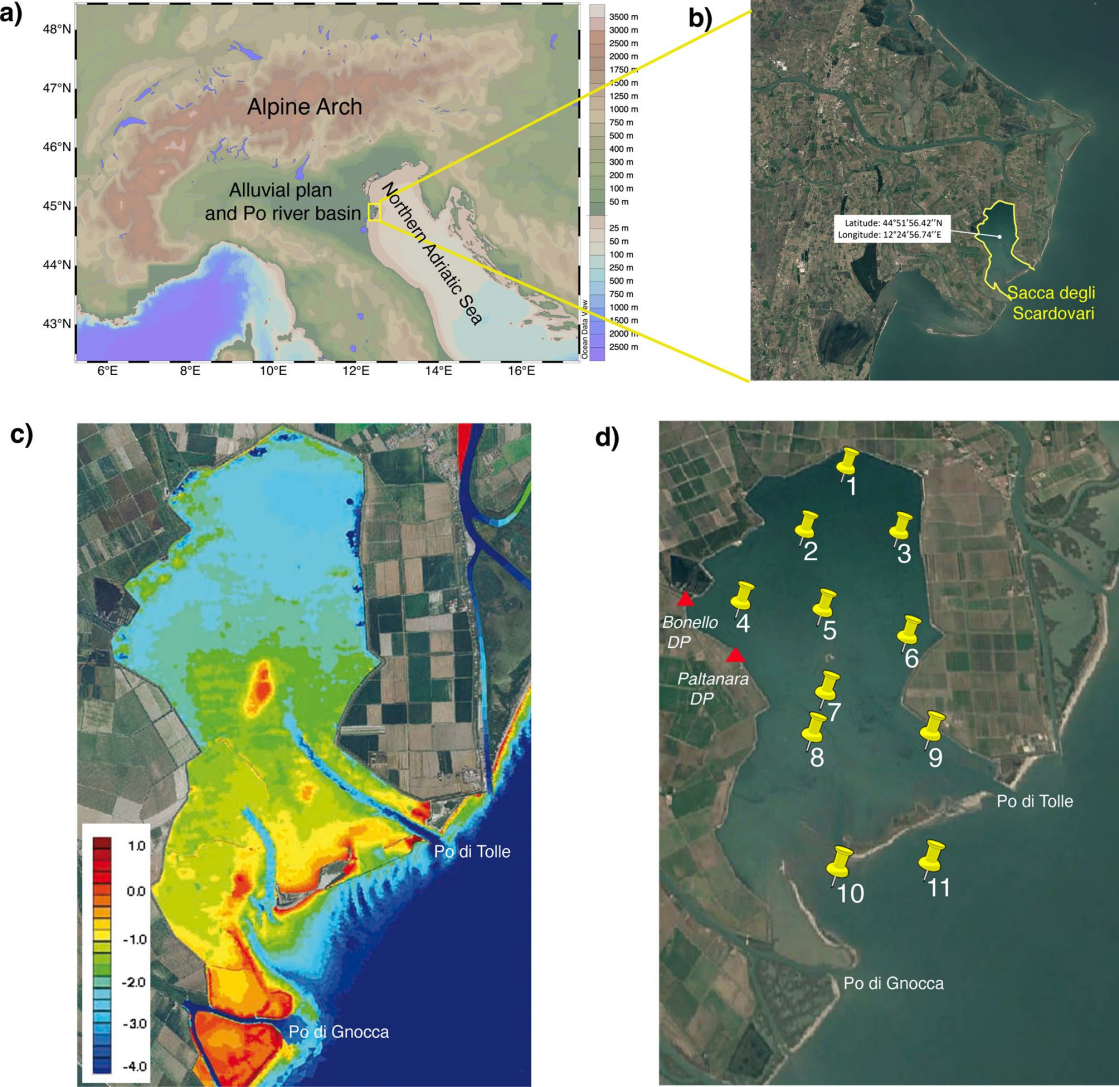
Geographical location of the study site. (**a**) The Alpine Arch and the Po alluvial plan with a colour scale indicating the soil elevation and bottom depth (Ocean Data View—odv.5.7.1—http://odv.awi.de); (**b**) Sacca degli Scardovari (SC) in the Po Delta (PD). Map data: Google, CNES/Airbus. Software used: Google Earth Pro and Adobe Photoshop; (**c**) bathymetric representation of the SC with a colour scale indicating the bottom depth, courtesy of D’Alpaos L.^22^; (**d**) map of sampling stations with dewatering pumps indicated by red triangles. The spatial positions of Sts 10 and 11 changed according to the dynamics of the sand bar. Map data: Google, CNES/Airbus. Software used: Google Earth Pro and Adobe Photoshop.

One of the macroscopic effects of ongoing climatic changes is the melting of global glaciers, which directly affects the spatial and temporal scales of the resulting runoff waters. Coastal deltaic lagoons are among the receiving systems whose ecological equilibria and exploitable resources are particularly sensitive to these changes^1–5^. Deltas are systems characterised by multiple interactions between terrestrial, freshwater, and marine forces that generate a highly dynamic environment threatened by floods, droughts, storm surges, and rising sea levels^6–13^. In the last century, natural processes and anthropogenic pressures have strongly affected the geomorphology and socioeconomic activities of many deltaic systems on Earth. The Po Delta (PD) represents the terminal part of an extensive alluvial plane occupying a large part of the Italian Peninsula crossed by the Po River, representing the collector of the entire alpine runoff waters from the Italian side of the Alpine Arch (Fig. 1a). The PD is a Man and Biosphere Reserve (https://en.unesco.org/biosphere/eu-na/delta-po) that extends over an area of 700 km^2^ ^14^, where five main Po River branches and a series of small branches feed seven highly productive coastal lagoons (Caleri, Marinetta, Barbamarco, Basson, Canarin, Scardovari, and Goro)^9^. The exceptional productivity of PD depends mainly on the high load of suspended organic matter (i.e., seston) and inorganic nutrients carried by the Po River^15–18^. In recent decades, the heavy reduction in the sediment load of the Po River and subsidence processes have triggered significant erosive phenomena along deltaic beaches, favouring saltwater intrusion with strong ecological alteration for both terrestrial and lagoon systems^19–27^. In this respect, shellfish farming activity in the PD is susceptible to all the ecological variations that deltaic environments tend to amplify^28^. The PD is one of the most critical sites for shellfish, mainly mussel, clam, and oyster, cultivation in Europe^29^, the yields of which are clearly declining, and this trend has been amplified by intense hydrographic and environmental crises that have occurred in the last few years^30^.

Among the biological communities, phytoplankton represent the first compartment to rapidly respond to environmental changes in marine environments and are the most digestible trophic source for seston filter feeders^31–38^. Furthermore, in PD basins with intensive shellfish aquaculture, phytoplankton are the primary producers, as macrophyte benthic assemblages are regularly extirpated to provide additional physical and functional space for the benthic clam *Ruditapes philippinarum* (Adams & Reeve, 1850)^18^. Despite its relevant role, scientific information regarding phytoplankton community dynamics in PD is still scarce, particularly regarding the levels at which environmental and biological processes play community and ecosystem roles, as well as climate change^15,18,39^.

The present work addresses the influence of the Po River flow rates on the phytoplankton community structure at the coastal lagoon of the PD, Sacca degli Scardovari (SC) and the potential impact of mussel filtration on these phytoplankton populations. We have considered the chemotaxonomic composition and size fractions as phytoplankton functional groups structuring the entire food web^40,41^, together with the total phytoplankton biomass, in relation to the physicochemical properties of the water column between April 2022 and May 2023.

## Results

### Salinity, temperature, and nutrient concentrations

**Fig. 2:**
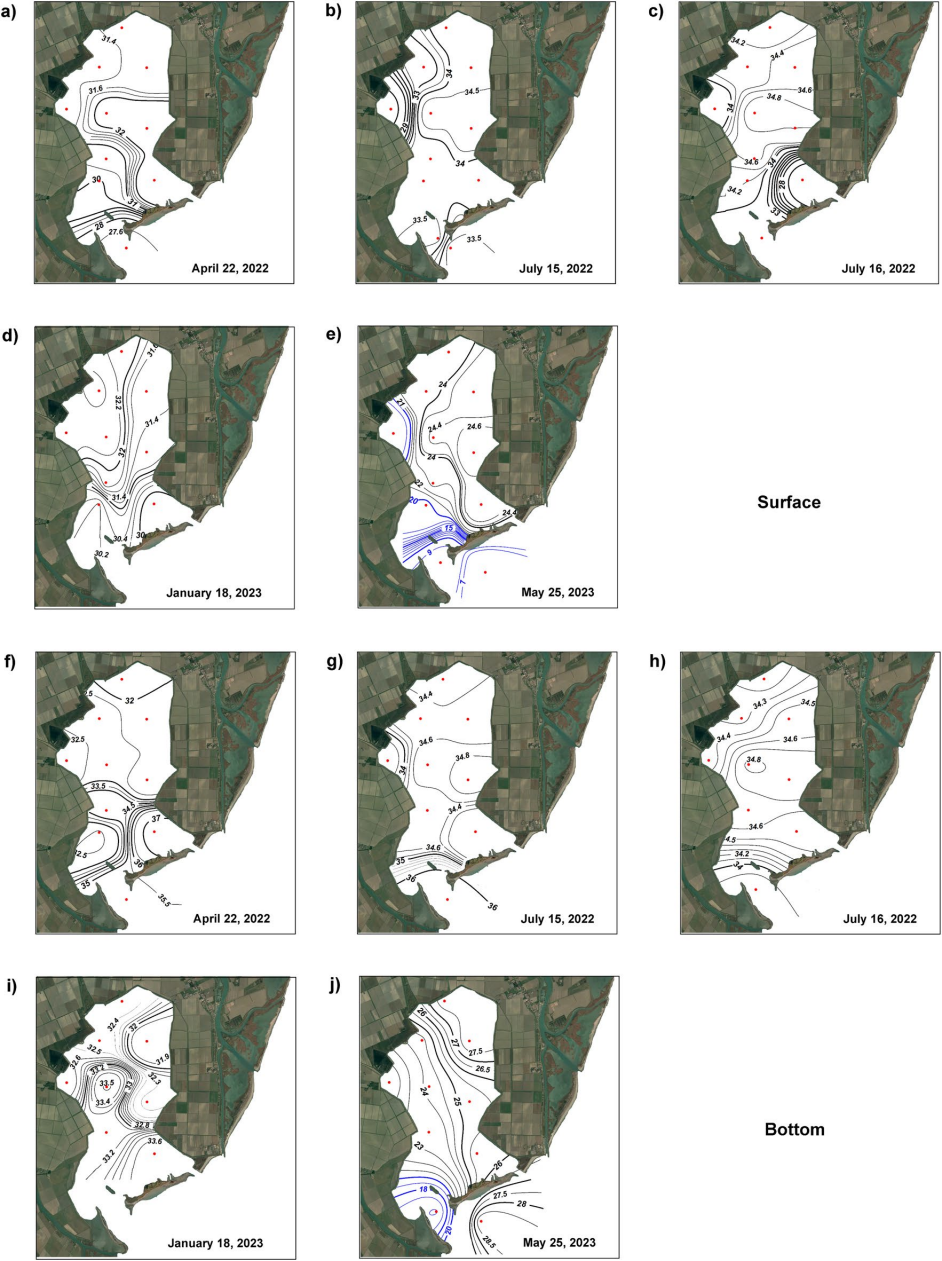
Spatial and temporal variations in salinity at the surface and bottom layers for each sampling period. 26 April 2022: (**a**) surface and (**f**) bottom; 15 July (1) 2022: (**b**) surface and (**g**) bottom; 16 July (2) 2022: (**c**) surface and (**h**) bottom; 18 January 2023: (**d**) surface and (**i**) bottom; 25 May 2023: (**e**) surface and (**j**) bottom. The blue lines indicate salinities < 20. Map data: Google, CNES/Airbus. Software used: Google Earth Pro and Adobe Photoshop. The interpolation of values was performed according to weighted-average gridding by Ocean Data View (odv.5.7.1—http://odv.awi.de).

The salinity distribution showed high spatiotemporal variability due to tidal inflow from the Adriatic Sea and, to a lesser extent, inputs from the dewatering pumping stations. In this regard, it must be considered that the Adriatic coastal water itself, which is influenced by Po River discharge^42,43^, already contains diluted waters. These two dilution dynamics generate a layer of relatively fresh and less dense water at the surface and a denser and saltier layer at the bottom. The spatial distribution of salinity in the two layers is shown in Fig. 2.

In April 2022, surface salinity values ranged between 27.56 (St 11) and 32.27 (St 6), with a gradient near stations facing the Adriatic Sea. At the bottom, the salinity ranged between 31.84 (St 1) and 37.05 (St 9), with a net gradient between the central and southern stations.

On July (1) 2022, a sharp salinity gradient was observed at the surface near the Bonello dewatering pump, with values ranging between 27.84 (St 4) and 34.83 (St 6). In the eastern and southern sectors, the salinities were relatively homogeneous (34.50 ± 0.3 and 33.60 ± 0.2, respectively). At the bottom, the salinity ranged between 33.73 (St 4) and 36.10 (St 11), with a weak gradient near St 4.

In July (2) 2022, a salinity gradient was observed at the surface in the southeastern sector, where salinities ranged between 27.37 (St 9) and 34.83 (St 6); a weak gradient was also observed near station 4, where salinities ranged between 33.74 (St 4) and 34.88 (St 5). At the bottom, the salinity was similar at all stations (~ 34.5), with a minimum of 33.84 observed at St 11.

In January 2023, the salinity at the surface ranged between 29.89 (St 9) and 32.42 (St 2), generating a gradient between stations 7 and 8. At the bottom, the salinities ranged between 31.81 (St 3) and 33.64 (St 9), with a weak gradient in the central sector.

In May 2023, the highest salinity variation in the overall sampled period because of the Po River flooding was observed (Fig. S1). At the surface, the salinities ranged between 6.11 (St 11) and 24.67 (St 6) for the southern sector, and the area near the Bonello dewatering pump was characterised by salinities lower than 20 (Fig. 2—blue line). In particular, two strong salinity gradients were observed: between Sts 10 and 11 and stations 9 and 8, where the salinities ranged from 6.11 to 24.5, and between Sts 4 and 5, where the values ranged between 18.63 (St 4) and 24.41 (St 5). At the bottom, the salinities were greater, producing a strong halocline at St 11, where the salinity ranged between 6.11 (surface) and 29.88 (bottom). In the northern sector, the salinities ranged between 28.01 ± 0.2 (Sts 1 and 3) and 24.59 ± 1 (Sts 2, 5, 6), while in the southern sector, the salinities were 29.88 (St 11), 25.99 (St 9), and 14.72 (St 10). A comparison between all values recorded at each station during the overall sampling period is reported in Fig. S2.

Unlike salinity, the temporal variation in temperature reflected the season cycle, although differences between the surface and bottom layers, as well as between the different subsectors of SC, were still observed (Fig. S3).

In April 2022, the surface temperature ranged between 14.39 °C (St 1) and 12.57 °C (St 11), while the bottom temperature ranged between 11.46 °C (St 9) and 14.69 °C (St 1), showing an increasing gradient moving from the southern to the northern sector.

In July (1) 2022 and July (2) 2022, the temperatures were rather homogeneous throughout the entire SC. In July (1), the temperatures ranged between 26.75 °C (St 4 and 9) and 27.31 °C (St 6) at the surface, while at the bottom, the temperatures ranged between 27.29 °C (St 6) and 26.7 °C (St 11). In July (2) 2022, the surface temperature ranged between 27.92 °C (St 3) and 27.22 °C (St 10), while the bottom temperature ranged between 27.13 °C (St 9) and 27.89 °C (St 3).

In January 2023, the lowest temperatures of the entire sampled period were observed, with surface temperatures ranging between 8.21 °C (St 1) and 9.55 °C (St 8) and values at the bottom ranging between 8.4 °C (St 2) and 10.53 °C (St 7).

In May 2023, the temperature showed a net gradient between the northern and southern sectors, with lower temperatures at stations facing the Adriatic Sea. At the surface, temperatures ranged between 19.19 °C (St 11) and 23.24 °C (St 3), while at the bottom, the temperatures ranged between 18.95 °C (St 11) and 23.48 °C (St 9), generating a strong gradient in the area facing the sea.

The temporal and spatial distributions of nutrient concentrations were influenced by the contributions of the waters of the Adriatic Sea on one side and the branches of the Po River, as well as by dewatering pumps on the other (Table S1).

In April 2022, the P-PO_4_ concentration ranged between 0.115 μmol/l (St 9, bottom) and 0.359 μmol/l (St 3, surface) and exhibited relatively high values near the dewatering pumps; the N-NO_3_ concentration ranged between 1.105 μmol/l (St 1) and 5.431 μmol/l (St 11), indicating relatively low values at the stations influenced by freshwater inputs. The Si-SiO_4_ concentration ranged between 0.941 μmol/l (St 11, bottom) and 2.644 μmol/l (St 5, bottom). The N/P ratio increased from the northern to the southern sector, ranging between 4.027 μmol/l (St 1) and 25.273 μmol/l (St 11).

In July (1) 2022, the P-PO_4_ concentration ranged between 0.112 μmol/l (St 11, bottom) and 0.701 μmol/l (St 6, surface), showing higher values in the central and southern sectors, especially near the dewatering pumps; the N-NO_3_ concentration was one order of magnitude lower than that in April 2022, especially at stations near the pumps, and ranged between 0.022 μmol/l (St 4, surface) and 0.580 μmol/l (St 1, bottom); the Si-SiO_4_ concentration ranged between 1.057 μmol/l (St 11, surface) and 5.782 μmol/l (St 2, bottom), showing higher concentrations in the central and northern sectors. The N/P ratio was particularly low (< 3) in the entire SC, ranging between 0.044 μmol/l (St 2, surface) and 3.053 μmol/l (St 11).

In July (2) 2022, the nutrient concentration was similar to that observed in July 2022, except for relatively low N/P ratios, which ranged between 0.061 μmol/l (St 10, surface) and 0.232 μmol/l (St 1, bottom). Higher P-PO_4_ concentrations were observed in the central sector, ranging between 0.226 μmol/l (St 7) and 0.599 μmol/l (St 6); the N-NO_3_ concentration was < 0.09 μmol/l in the entire area. The Si-SiO_4_ concentration ranged between 2.440 μmol/l (St 10, surface) and 8.865 μmol/l (St 9, bottom), showing a high concentration in the central-western sector.

In January 2023, the N-NO_3_ concentration ranged between 2.007 μmol/l (St 2) and 9.788 μmol/l (St 9), the P-PO_4_ concentration ranged between 0.189 μmol/l (St 2, surface) and 0.630 μmol/l (St 9, surface), and the Si-SiO_4_ concentration ranged between 2.088 μmol/l (St 5) and 4.078 μmol/l (St 4), reaching a maximum near the pumps. The increase in N-NO_3_ concentration led to a rise in N/P ratios compared to those in July, especially near the pumps and in the southern sector, with values ranging from 4.434 μmol/l (St 7, bottom) to 26.716 μmol/l (St 4).

In May 2023, the N-NO_3_ concentration notably increased, unlike the P-PO_4_ concentration. In particular, the N-NO_3_ concentration ranged between 4.821 μmol/l (St 1, bottom) and 24.190 μmol/l (St 6, bottom), showing high point variability, while the P-PO_4_ concentration ranged between 0.046 μmol/l (St 1) and 0.451 μmol/l (St 6, bottom). These changes led to an N/P ratio > 29 in the entire SC, with values ranging between 29.5 (St 4) and 103.693 (St 1). The Si-SiO_4_ concentration ranged between 5.666 μmol/l (St 9) and 24.545 μmol/l (St 2, surface).

### Total phytoplankton biomass and size classes

**Fig. 3:**
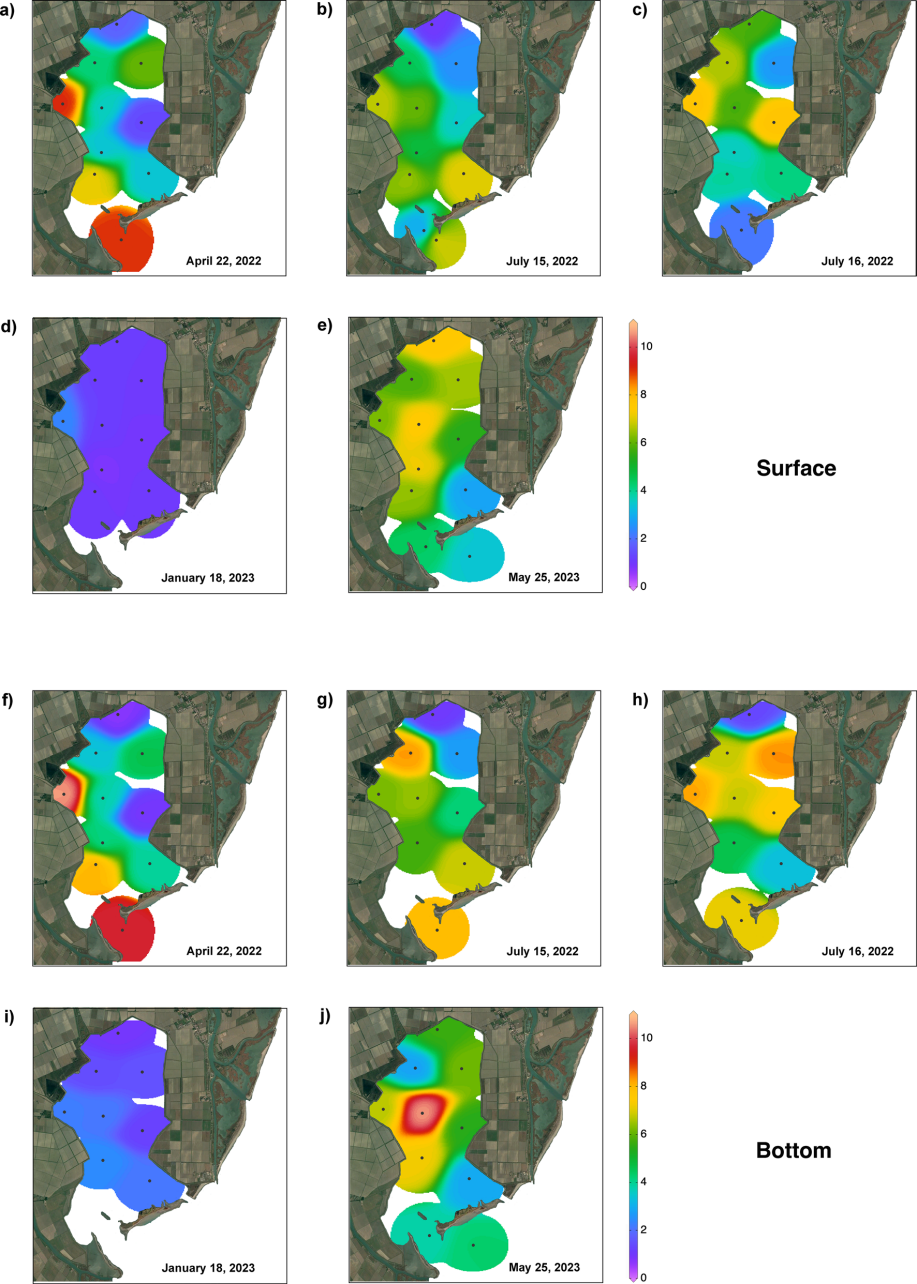
Spatial and temporal variation in total phytoplankton biomass (Chl a, μg/l) in the surface and bottom layers for each sampling period. 26 April 2022: (**a**) surface and (**f**) bottom; 15 July (1) 2022: (**b**) surface and (**g**) bottom; 16 July (2) 2022: (**c**) surface and (**h**) bottom; January 18, 2023: (**d**) surface and (**i**) bottom; 25 May 2023: (**e**) surface and (**j**) bottom. Map data: Google, CNES/Airbus. Software used: Google Earth Pro and Adobe Photoshop. The interpolation of values was performed according to weighted-average gridding by Ocean Data View (odv.5.7.1—http://odv.awi.de).

**Fig. 4:**
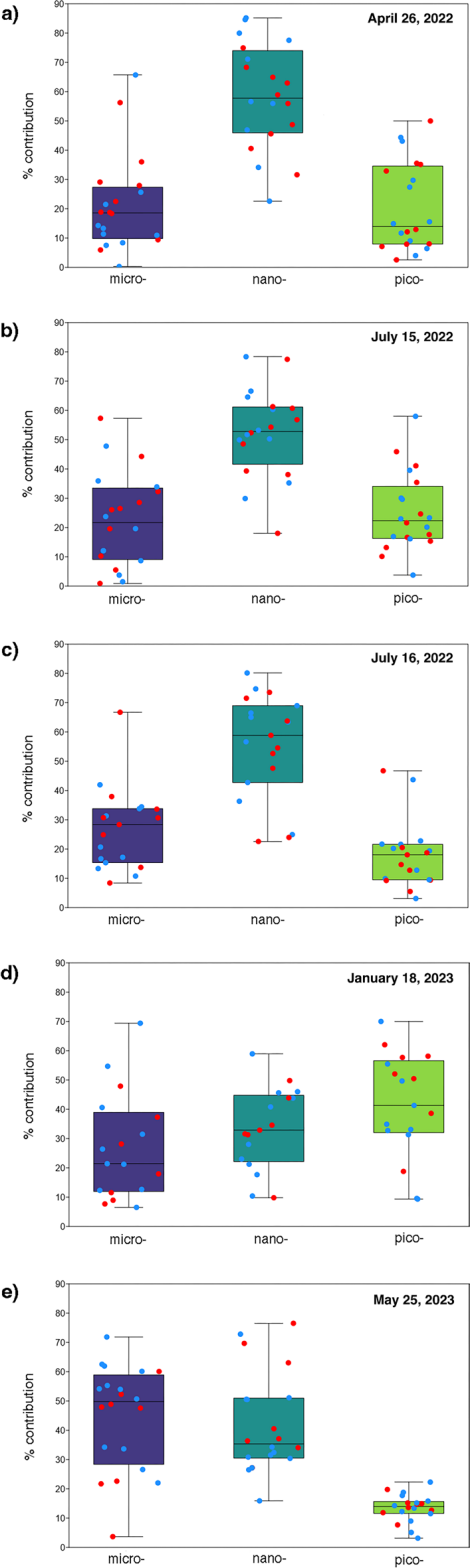
Box and jitter plot with percentage contributions of the micro, nano- and pico-phytoplankton size classes in the surface (blue dots) and bottom (red dots) layers on (**a**) 26 April 2022; (**b**) 15 July (1) 2022; (**c**) 16 July (2) 2022; (**d**) January 18, 2023; and (**e**) 25 May 2023.

The role of the phytoplankton community, which drives a major part of the lagoon food web, has been studied in terms of total biomass and its functional group composition. The functional group chosen for further analysis of phytoplankton biomass was the size class, as this parameter is a more direct tool for evaluating consumption by filter-feeding shellfish, with the intent of assessing the strength of the filtration activity based on the size structure of the plankton community. The variation in the phytoplankton biomass (chlorophyll a (Chl a) concentration) in the surface and bottom layers is shown in Fig. 3, and the contributions of the size classes are summarised in Fig. 4. Overall, the phytoplankton biomass showed uneven distribution, with a net gradient between the northern and southern sectors of the SC. In general, relatively high values were observed near the Bonello dewatering pump and at stations facing the sea.

In April 2022, the Chl a concentration reached 9.16 μg/l (St 4), and two minima were observed at Sts 1 (1.77 μg/l) and 6 (1.38 μg/l). At the bottom, values ranged between 9.69 μg/l (St 4) and 1.61 μg/l (St 1). The nanofraction represented approximately 60% of the community at the surface, exceeding 85% at stations facing the sea. The pico- and microfractions showed similar mean values (18 and 21%, respectively). Microfraction variation showed uneven distribution, while picofraction variation was more abundant in the northern sector, where percentages reached 44% (St 1). At the bottom, the variation trends were similar, with the nanofraction dominating the community (mean 55%) and the micro- and picofractions representing 24% and 20%, respectively.

In July (1), the pattern of distribution was similar to that in April 2022 in both the surface and bottom layers, although the concentration of Chl a was lower, ranging between 7.68 μg/l (St 11) and 1.02 μg/l (St 1) at the surface and between 7.55 μg/l (St 2) and 0.96 μg/l (St 1) at the bottom. The nanofraction dominated the community (mean 57%), reaching a maximum of 77% (ST. 10); micro- and nanofractions accounted for 18% and 25%, respectively, of the total phytoplankton biomass. At the bottom, the trend was similar; the nanofraction dominated the community (mean 50%), and micro- and picofractions had mean percentages of 25% and 24%, respectively.

In July (2) 2022, strong differences were observed between the bottom and surface layers at Sts 3 and 11, with a marked vertical Chl a gradient. The values ranged between 7.75 μg/l (St 4) and 2.14 μg/l (St 10) at the surface and between 8.40 μg/l (St 3) and 1.32 (St 1) at the bottom. The nanofraction dominated the community (mean 58%), reaching the highest values between Sts 1 and 4. The micro- and picofractions showed mean percentages of 23% and 19%, respectively, with the micro- and picofractions being more abundant at stations facing the sea. At the bottom, the trend in fraction variations was similar. The nanofraction dominated the community (mean 50%), the microfraction showed a mean percentage of 31%, and the picofraction represented only 18% of the phytoplankton, reaching higher percentages in the northern sector.

In January 2023, low concentrations of biomass in the entire SC in both the surface and bottom layers were observed. At the surface, the Chl a concentration reached a maximum of 2.19 μg/l (St 4) and a minimum of 0.72 μg/l (St 7); at the bottom, the Chl a concentration ranged between 2.48 μg/l (St 7) and 0.97 μg/l (St 1). The picofraction dominated the community in the surface layer. This group showed a mean concentration of 45%, reaching 70% at St 2. The mean values of the micro- and nanofraction concentrations were 25% and 30%, respectively. At the bottom, the pico- and nanofractions had the same mean percentage (37%), with different distributions. The picofraction was more abundant in the northern sector, where its concentration exceeded 50% (Sts 1, 2, 3), and the nanofraction was more abundant at stations facing the sea, reaching 45% (Sts 8, 9). The mean percentage of the microfraction was 26%, which was more abundant in the central sector.

In May 2023, the highest Chl a concentration of 10.60 μg/l was observed at the bottom of station 5. At the surface, values of ~ 7.5 ± 0.2 μg/l were observed at Sts 1, 5, and 7, generating a clear E‒W gradient; the minimum value was 2.87 μg/l (St 9). The bottom showed a similar pattern (except for St 2), with values ranging between 10.60 and 2.95 μg/l at St 9. At the surface, the microfraction represented, on average, 48% of the community and was more abundant in central-northern sectors, where it reached a maximum concentration of 72% (St 1). The mean percentage of the nanofraction reached 38% at the station facing the sea; the nanofraction represented only 14% of the community. At the bottom, the nanofraction dominated the community, with a mean concentration of 52%. The microfraction showed a mean concentration of 30%, exceeding 40% at Sts 1 and 5, while the picofraction represented 18% of the community.

### Chemotaxonomical functional composition

**Fig. 5:**
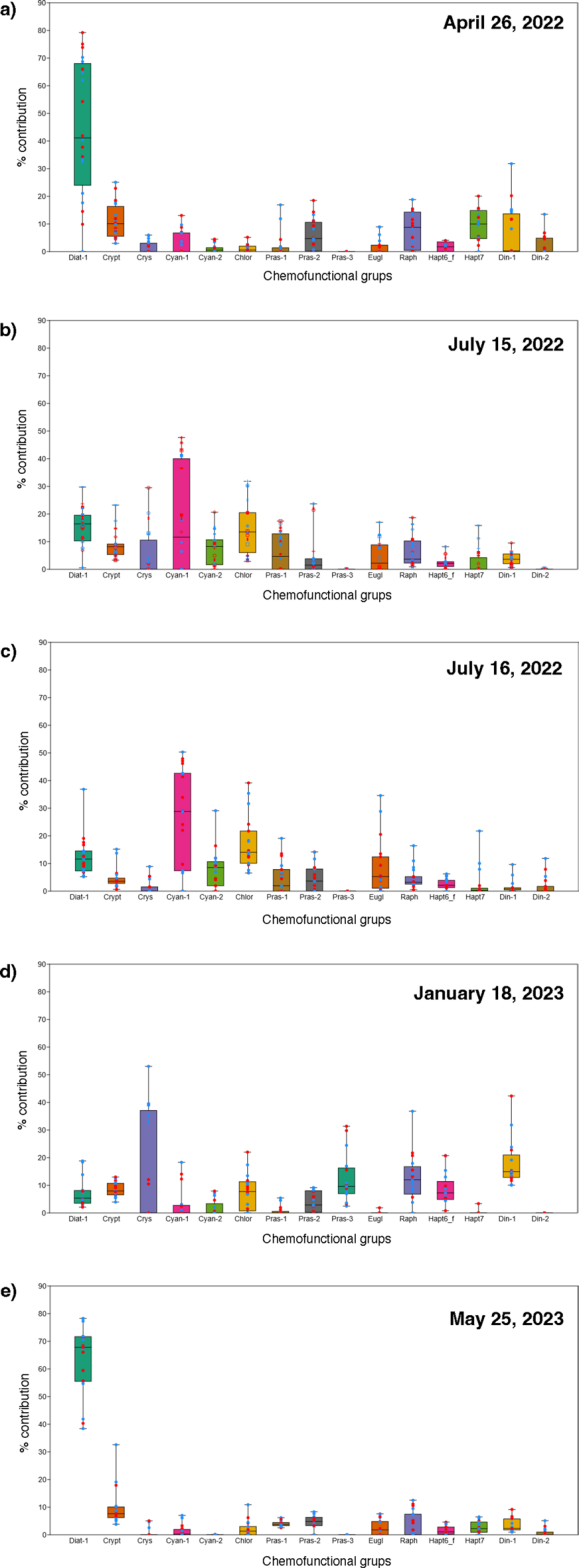
Box and jitter plot with percentage contributions of phytoplankton chemiofunctional groups in the surface (blue dots) and bottom (red dots) layers. (**a**) 26 April 2022; (**b**) 15 July (1) 2022; (**c**) 16 July (2) 2022; (**d**) 18 January 2023; and (**e**) 25 May 2023. Diat-1: diatoms; Crypt: cryptophytes; Crys: crysophytes; Cyan-1: cyanophytes with a low zeaxanthin/Chl a ratio; Cyan-1: cyanophytes with a high zeaxanthin/Chl a ratio; Chlor: chlorophytes; Pras-1: prasinophytes; Pras-2: prasinophytes with a high MGDVP:Chl a ratio; Pras-3: prasinophytes containing prasinoxanthin; Eugl: euglenophytes; Raph: raphidophytes; Hapt6_f: haptophytes; Hapt7: haptophytes containing MGDVP; Din-1: dinoflagellates containing peridinin; Din-2: dinoflagellates.

In April 2022, the phytoplankton community was strongly dominated by diatoms in both the surface (mean 41%) and bottom (mean 48%) layers, exceeding 75% on the bottom of the northern sector (Fig. 5). In particular, the surface layer was characterised by a relatively greater contribution of dinoflagellates (Sts 1, 2, 3, 5), crysophytes (Sts 7, 8, 9, 10, 11) and euglenophytes (Sts 6, 7) compared to the bottom layer. Chlorophytes were relatively abundant (mean 3 ± 2%) at Sts 5, 6, and 7 near the Bonello pumping station.

In July 2022, a net decrease in diatoms was observed in the entire SC in both layers. The phytoplankton community exhibited a more heterogeneous composition, with no dominance of single groups.

In July (1), chlorophytes were the most abundant group (mean 20%) at the surface, especially at northern stations 1, 3, and 4, near the Bonello pumping station, where they reached ~ 30%. Diatoms represented, on average, 15% of the community and were less abundant in the central sector (minimum of 0.5% at St 7). Cryptophytes and cyano-2 represented, on average, ~ 10% of the community and were homogeneously distributed in the SC, except for a maximum of cryptophytes (23%) at St 2. At the bottom, St 3 was characterised by high percentages of cyano-2, chlorophytes and raphidophytes (with a mean of ~ 20% each), while cyano-1 was more abundant in the central-southern sector (Sts 5, 6, 7, 9, 11), where percentages reached 47%; dino-1 was more abundant at stations facing the Adriatic Sea and Bonello pumping station than at other stations.

In July (2) 2022, a net increase in cyano-1 was observed over the entire area, especially at the surface layer, where the group showed a mean of 30% and reached percentages of up to 50% at station 4 near the Bonello pump. Chlorophytes were the second most abundant group in both layers, with relatively high percentages at the bottom, where they reached a maximum of 39% at St 2; at the surface layer, the group was relatively more abundant in the central area (Sts 3, 4, 5, and 6). Prasino-1 was more abundant (> 10%) in the surface layer (Sts 1, 4, 6, and 7) than in the bottom layer.

In January 2023, the community was dominated by dinoflagellates, crysophytes, prasinophytes-3, and raphidophytes, although with different distribution patterns. Crysophytes dominated the surface in the northern sector (Sts 1, 2, 3, and 4), where percentages between 35 and 50% were reached; at the bottom, the percentage of this group was four times lower. The percentages of dinoflagellates, prasinophytes-3, and raphidophytes at the surface were 16%, 13%, and 12%, respectively, while at the bottom, the percentages of dinoflagellates, prasinophytes-3, and raphidophytes were 19%, 12% and 11.2%, respectively.

In May 2023, the surface and bottom layers were strongly dominated by diatoms, with a mean percentage of ~ 63% and a maximum exceeding 70% at stations 1, 2, 6, and 8 at the surface. The minimum percentage of diatoms (40%) was observed in the bottom layer at station 10. At the surface, cryptophytes were the second most abundant group, reaching relatively higher percentages between Sts 7 and 11 (9–32%). Dino-2 was detected only in the bottom layer at Sts 10 (2%) and 11 (5%).

### Network plot analysis

**Fig. 6:**
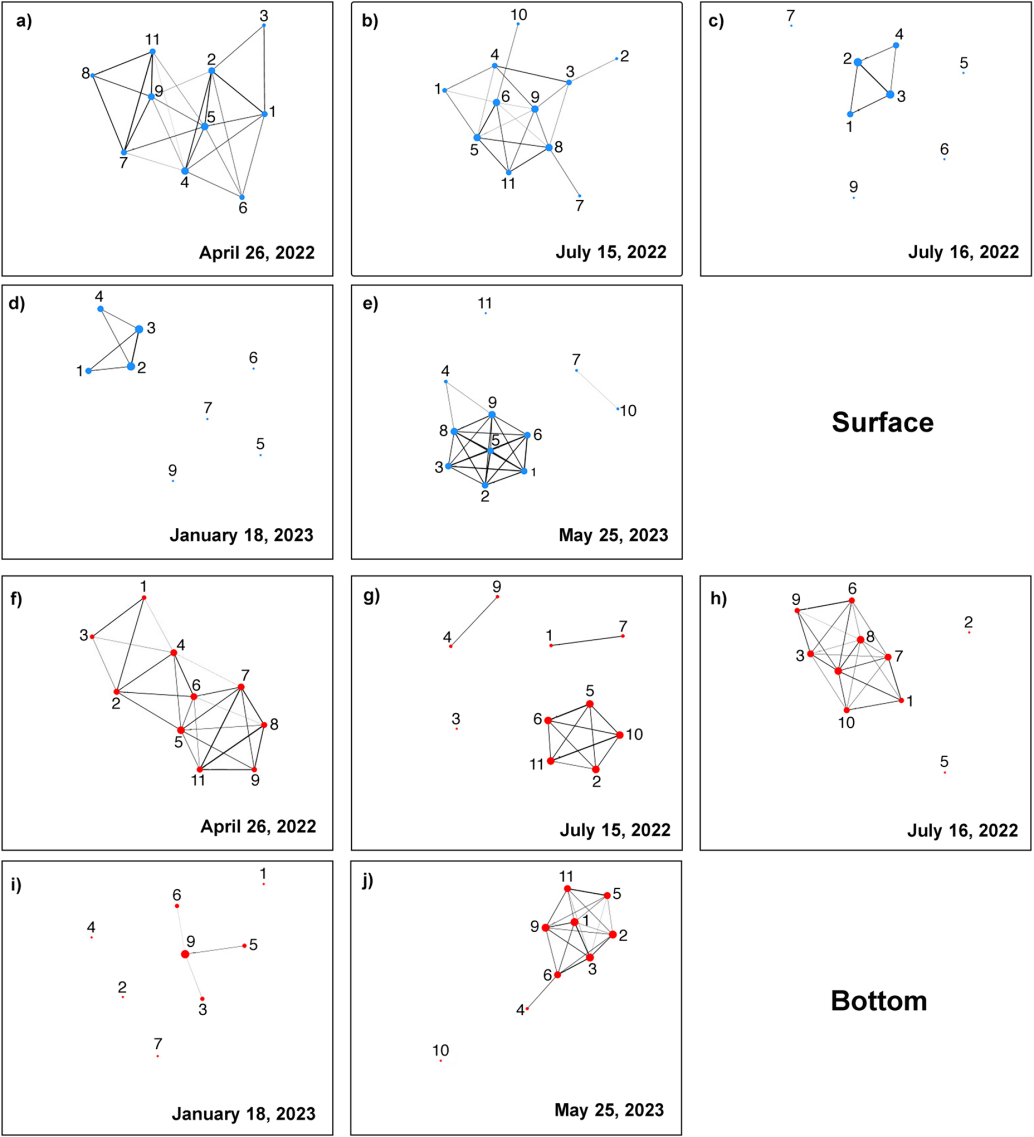
Network plot analysis of the chemofunctional composition of phytoplankton at the surface (blue dots) and bottom (red dots) stations for each sampling period. 26 April 2022: (**a**) surface and (**f**) bottom; 15 July (1) 2022: (**b**) surface and (**g**) bottom; 16 July (2) 2022: (**c**) surface and (**h**) bottom; January 18, 2023: (**d**) surface and (**i**) bottom; 25 May 2023: (**e**) surface and (**j**) bottom. To determine the degree of similarity between stations, a network plot analysis was developed based on the Bray‒Curtis similarity index, in which the degree of similarity between different stations was established based on the chemofunctional composition of the communities in the surface (blue dots) and bottom (red dots) layers. A 50% cut-off value was used to show only edges between nodes that were more than 50% similar. The nodes were arranged based on the Fruchterman-Reingold algorithm^64^, and stations with a similar composition were connected with a thick black line.

The chemofunctional diversity described thus far produces network plots with clear temporal and spatial variability in both the surface and bottom layers (Fig. 6). The plots represent the influence of the factors driving the structural variations in environmental and ecological terms of the lagoon water masses, as they respond to different inputs that can produce effects detectable at large spatial but short temporal scales, such as river floods.

In April 2022, two groups formed by Sts 7, 8,9, and 11 (showing greater similarity, as highlighted by the thick black line) and Sts 1, 2, 4, and 5 were observed. In the bottom layer, Sts 7, 8, 9, and 11 still showed greater similarity, although the black lines also connected Sts 1, 3, 2, 4 and 6.

In July (1) 2022, a similar composition was observed for Sts 3 and 4 and Sts 6, 5, 11, and 8. At the bottom, thick black lines connect Sts 4 and 9 and Sts 1 and 7, forming two isolated groups. Sts 6, 5, 11, 10 and 2 formed a single group, with the greatest similarities observed between Sts 6 and 5 and 11 and 10.

In July (2) 2022, the only group identified consisted of Sts 1, 2, 3 and 4, with Sts 2 and 3 showing a high degree of similarity. At the bottom, Sts 2 and 5 were not connected to the other stations. Within the only group present, a high degree of similarity was observed between Sts 1, 4, 3, 9, and 6 and Sts 1 and 7.

In January 2023, Sts 1, 2, 3, and 4 still presented a high degree of similarity, as observed in July (2). In the bottom layer, the only stations with similar compositions were Sts 9, 5, 6, and 3.

In May 2023, the stations facing the sea appeared to have completely dissociated from the other stations (with a weak connection between Sts 7 and 10). The other stations formed a single group, in which St 4 had a different composition compared to the others. At the bottom, St 10 appeared isolated, and among the large groups formed by all stations, St 4 showed a single connection with St 6.

### Mesocosm experiment

**Fig. 7:**
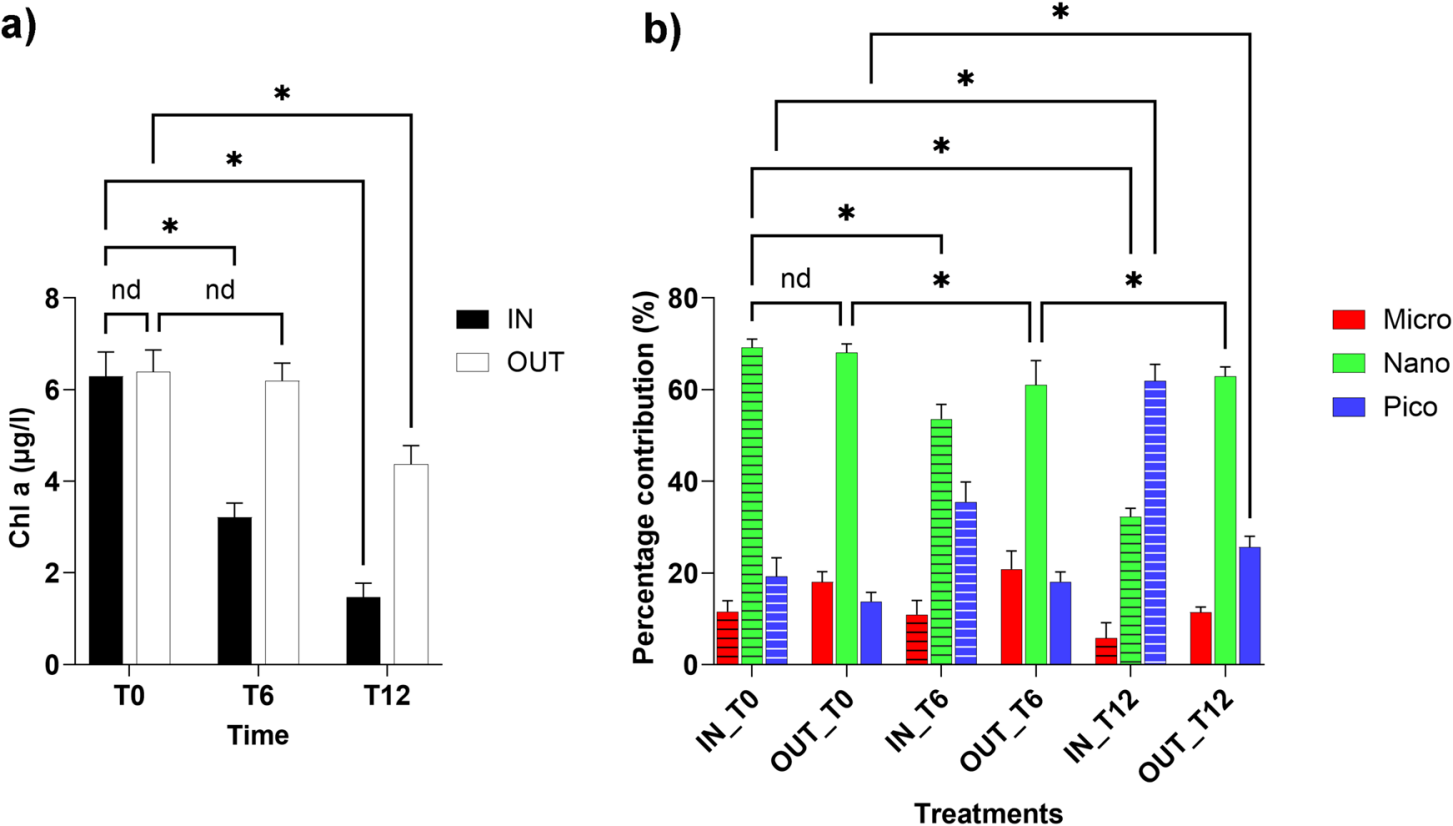
Histograms reporting the temporal variations in total phytoplankton biomass (**a**) and percentage contributions of micro, nano- and pico-size classes (**b**) at three different timepoints (hours) in the in situ mesocosm mussel filtration experiment. T0: start, T6: after 6 h; T12: after 2 h.

The strong variations in Chl a concentration observed in the chamber highlighted the effects of mussel filtering activity on phytoplankton standing crops (Fig. 7, Table S2). After 12 h of mussel filtration, the concentration of phytoplankton biomass within the three chambers strongly decreased, with mean values of 6.28 ± 0.53 μg/l (T0), 3.16 ± 0 0.30 μg/l (T6) and 1.46 ± 0.31 μg/l (T12). Outside the chamber, the mean concentrations were similar at the first two timepoints, specifically, 6.38 ± 0.48 μg/l (T0) and 6.19 ± 0.38 μg/l (T6), with a slight decrease at T12, when the concentration reached 4.37 ± 0.40 μg/l.

These variations were accompanied by a net shift in the size structure composition within the chamber, where the nanofraction proportion strongly decreased, unlike the picofraction, the relative contribution of which almost tripled. The concentration ranged from 69.15 ± 0.81% (T0) to 32.24 ± 1.5% (T12) for the nanofraction and from 19.27 ± 0.94% (T0) to 61.93 ± 2.16% (T12) for the picofraction. The concentration of microfraction ranged between 11.57 ± 0.57% (T0) and 5.82 ± 0.76% (T12).

Outside the chamber, in free water, the nanofraction concentration showed similar percentages at the different timepoints, with values of 68.12 ± 1.45% (T0), 61.12 ± 09% (T6), and 62.91 ± 1.10% (T12); the picofraction showed a weak increase, ranging from 15.80 ± 1.15% (T0) to 25.66 ± 0.69% (T12); and the microfraction ranged from 16.05 ± 0.62% (T0) to 1.42% (T12).

### Statistical analyses

Two-way permutational multivariate analysis of variance (PERMANOVA) results indicated the presence of significant temporal and spatial variations within SC. The magnitude of these changes mainly depended on the sampling period (F 36.780, *p* < 0.001) and, to a lesser extent, the location of the station (F 6.692, *p* < 0.001) in the northern (Sts 1, 2, 3), central (Sts 4, 5, 6, 7) and southern (Sts 8, 9, 10, 11) sectors of the lagoon (Table 1).

**Table 1:** Two-way permutational multivariate analysis of variance (PERMANOVA) to evaluate the extent of statistically significant differences between SC sectors (northern, central, and southern) and the seasonal variability.

Permutation N:	9999				
Source	Sum of sqrs	df	Mean square	*F*	*p*
Sector	0.39802	2	0.1999	6.692	0.0001
Month	4.37501	4	1.0938	36.780	0.0001
Interaction	0.77070	8	0.096337	3.240	0.0001
Residual	2.40873	81	0.029737		
Total	7.95250	95			

Sts 1, 2, 3 were grouped into sector 1, Sts 4, 5, 6, 7 into sector 2, and Sts 8, 9, 10, 11 into sector 3. The Bray–Curtis index was used as a similarity index. Significance was computed by permuting the group membership, with 9999 replicates.

**Fig. 8:**
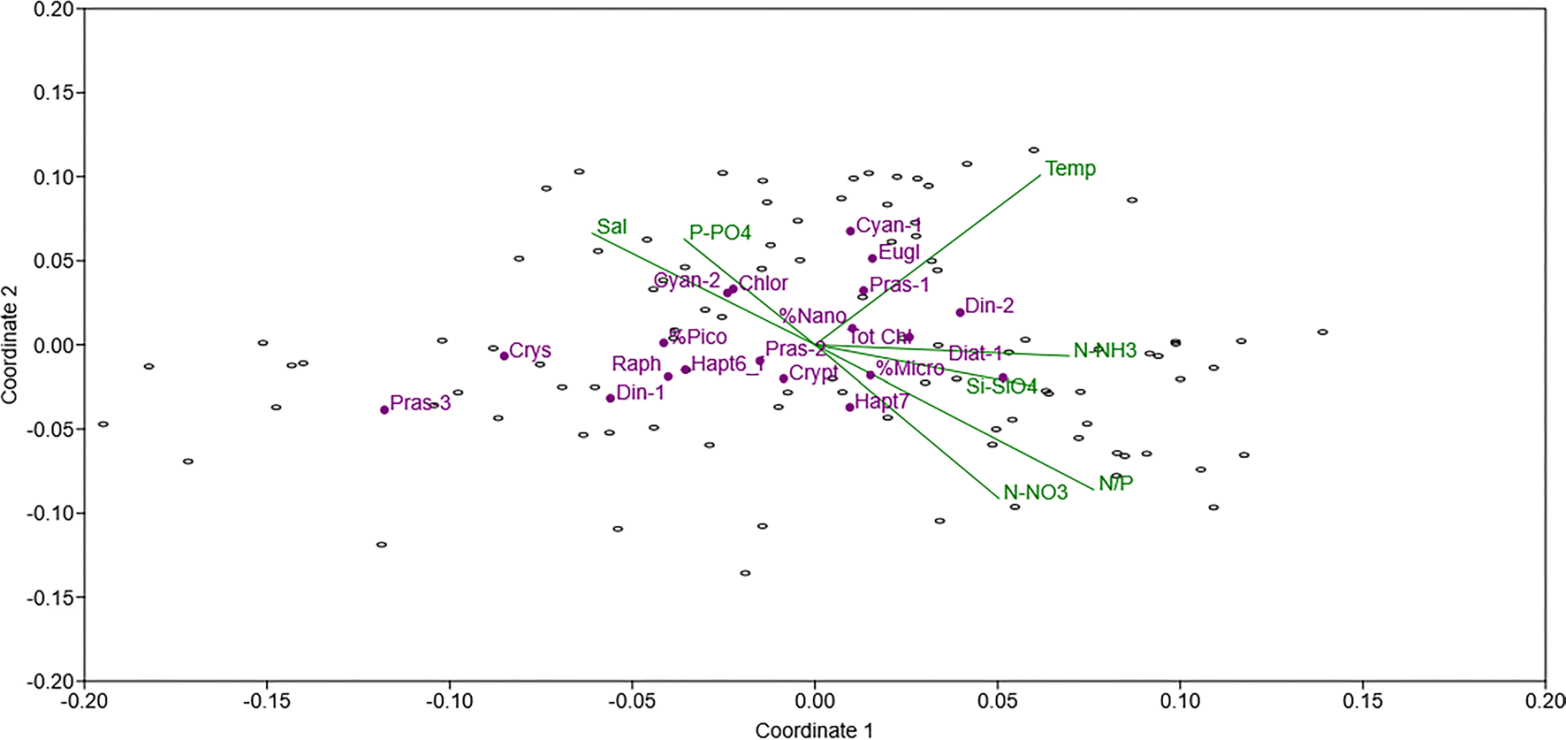
Nonmetric multidimensional scaling (NMDS) was performed based on a distance matrix computed with the Bray‒Curtis dissimilarity index. The environmental variables (Temp: temperature; Sal: salinity; N-NO_3_, Si-SiO_4_, P-PO_4_ and N-NH_3−_ concentrations and the N/P ratio) are reported in green and are not included in the ordination; the correlation coefficients between each environmental variable and the NMDS scores are presented as vectors from the origin (Stress: 0.25. Axis 1: 0.454; Axis 2: 0.2165). Tot Chl a: total phytoplankton biomass, %Micro: microphytoplankton, %Nano: nanophytoplankton, Pico%: picophytoplankton. Diat-1: diatoms; Crypt: cryptophytes; Crys: crysophytes; Cyan-1: cyanophytes with a low zeaxanthin/Chl a ratio; Cyan-1: cyanophytes with a high zeaxanthin/Chl a ratio; Chlor: chlorophytes; Pras-1: prasinophytes; Pras-2: prasinophytes with a high MGDVP:Chl a ratio; Pras-3: prasinophytes containing prasinoxanthin; Eugl: euglenophytes; Raph: raphidophytes; Hapt6_f: haptophytes; Hapt7: haptophytes containing MGDVP; Din-1: dinoflagellates containing peridinin; Din-2: dinoflagellates.

Nonmetric multidimensional scaling (NMDS) (Fig. 8), in which environmental variables were not included in the ordination system, highlights the role of salinity in the ecology of SC, especially regarding the P-PO_4_ and N-NO_3_ distributions and the related N/P ratios, which exceeded 90 during flood waves and decreased to 0.07 during the drought period in summer. Under relatively high salinity, cyano-2 (cyanophytes with a high zeaxanthin/Chl a ratio) and chlorophytes almost overlapped, suggesting a synergistic response. The relatively greater contribution of small size groups, such as prasinophytes, crysophytes and dino-1 (dinoflagellates containing peridinin), observed in January 2023 was highlighted by their relation to temperature, while the contributions of cyanophytes and euglenophytes were relatively greater under high temperature (July 2022). Dino-2 (dinoflagellates) made greater contributions under relatively high levels of N-NH_3_ and temperature. Diato-1 (diatoms) overlapped with the Si-SiO4 concentration (coordinate 1:0.06) and, together with the microfraction proportion (%) and hapt7 (haptophytes containing MGDVP), were subjected to more dilution dynamics. The total phytoplankton biomass did not overlap with any variable but was grouped near the microfraction, nanofraction and diat-1 proportions, indicating a similar ecological response pattern.

## Discussion

This study aims to provide additional information on a coastal transitional system of crucial importance at the natural, cultural, and social levels in the Mediterranean Sea sector, namely, the PD, where bivalve farming activities are among the most important in Europe.

From an ecological perspective, bivalve aquaculture provides not only provisioning but also numerous other ecological services, such as regulative, maintenance, and supporting services, affecting ecosystem stability^44–46^. In our case, the above system is dominated by the influence of the Po River, with temporal variations in the volume of the contributed waters, exerting strong control over the trophodynamics of the SC. Furthermore, the effects of ongoing climatic changes on the littoral systems of the Adriatic Sea can be monitored through the direct influence of alpine ice retreat waters on the hydraulic regime of the Po River. As the Po River collects the drainage waters of the Italian side of the entire Alpine Arch, the phytoplankton dynamics reported here represent a useful baseline for further addressing issues of climatic changes affecting lagoon ecology.

The documented history of the SC within the pronounced environmental dynamics of the northern Adriatic coast allows for the tracing and disentangling of the complex interactions of natural and anthropogenic influences^22,47^. Notably, any study aimed at evaluating the ecological status and, hence, the carrying capacity of a system, provides the basic information necessary for every management activity^3,28^. Our research plan has been shaped considering the complexity of the SC system, whose environmental conditions are, on one hand, under the influence, in its inner part, of the water management of intensive agricultural activity on a large territorial belt surrounding the basin; on the other hand, by communication with marine waters, which are, in turn, affected by the high dynamics of the sand bar separating the lagoon from the sea and governing the influence of tidal cycles^22^. Overall, our data allowed for the identification of distinct ecological subsectors in the SC, with high scale-related temporal and spatial variability (Table 1). In particular, the distribution of phytoplankton biomass follows north–south and west–east gradients, depending on allochthonous inputs and shellfish filtration activities.

The highest concentrations of phytoplankton biomass were generally observed at stations facing the sea and dewatering pumping stations, where large fluctuations in salinity and nutrient concentrations were observed. In fact, diatoms were the dominant group in the presence of intense Po River inputs, which was the main driver of salinity distribution, while under a minimum river flow rate, the community showed a more heterogeneous composition with a greater contribution of chlorophytes and haptophytes.

The combined action of the factors driving the structural variations in environmental and ecological terms of the water masses affects the phytoplankton chemocomposition, which is detectable at large spatial but short temporal scales (Fig. 6).

In January, with low environmental temperatures and low light availability, the concentration of phytoplankton biomass was up to one order of magnitude lower than that in other seasons, with a greater contribution from dinoflagellates and prasinoxanthin-containing prasinophytes. Although tidal inflows represent the main factor driving the circulation and mass water exchanges between the lagoon and Adriatic Sea waters that are directly affected by Po River inputs^22^, the ecological effects of the pumping station are relevant in trophic terms, providing nutrient-rich inputs as well as living and dead particulate matter, generating a highly productive sector with higher densities of phytoplankton of large-sized classes (micro- and nanophytoplankton).

High concentrations of N-NO_3_ were also observed in relation to the incoming Po River inputs during the periods of higher floods. These factors exert strong control on the overall hydrographic and ecological dynamics of the SC (Fig. 8). In fact, the N/P ratio appeared to be > 90 during the flood wave and < 0.07 in summer during the drought period. The distribution of phytoplankton biomass, which is mainly dominated by the nanosize class (2–20 μm), is related to nonlinear freshwater inputs, and its structure is influenced by the filter-feeding activity of shellfish and water inputs according to their different origins.

The dewatering pumps, located on the northwestern shore, collect the surrounding agricultural waters, while the Po River branches represent the collectors of the entire Alpine Arch and human activities occurring throughout the whole alluvial Padanian Plane. The large biomasses of filter-feeding organisms not subject to collection and commercial consumption by humans deserve particular attention, as they cover the submerged fringe of the northwestern sector (mainly oysters), whose filtering activity certainly contributes to modifying the phytoplankton community structure in unpredictable ways due to the absence of any previous abundance estimates. The large, suspended biomasses of mussels exert a strong selective influence on the phytoplankton functional group composition. The mesocosm experiment revealed that nanophytoplankton largely support mussel production, highlighting the importance of this size group for the general food web economy of the lagoon. In fact, the filtration activity within the experimental chamber led to a decrease in Chl a concentration from 6.28 to 1.46 μg/l over 12 h; within this variation, the nanofraction proportion decreased, while that of the picofraction subsequently increased (Fig. 7). In this respect, reducing competition for food among phytoplankton filter feeders is an optimisation of the shellfish farming process with respect to the wall productive processes of the lagoon.

An important element in the ecology and economy of Scardovari is the high population density of the blue crab (*Callinectes sapidus*) (Table S4). This species is among those considered reliable indicators of climatic change, highly impacting ecosystem services and related economic revenues^48–51^. Its population has increased to such a rate to strongly affect all aquacultural activities. It is well documented that the crab is an efficient predator that acts on all macroscopic components of the food web^52–54^. In Scardovari, the cultivated species mussel (*Mytilus galoprovincialis*), clam (*Ruditapes philippinarum*) and oyster (*Crassostrea gigas*) have been heavily impacted. As a consequence, commercial biomass has dramatically decreased (Table S4), while the blue crab is exploiting all available biological resources. In addition, because this species is important to the dietary habits of local and broad consumer communities, management is forced to collect relevant, albeit insufficient, quantities of the crustacean to be channelled for waste treatment, with additional economic losses.

Considering the profound changes caused by climate change in the PD and Po River and its deltaic system, we believe that understanding the mechanisms regulating phytoplankton community structure and dynamics is a crucial step for ensuring rational shellfish farming in the area. Furthermore, economically important aquaculture activities are still carried out in most Mediterranean transitional water sites, and such activities are in extreme need of a sufficient database to implement any production activity and, in all cases, a fundamental point for preserving the enormous value of transitional waters as territorial assets. The rich and complex environmental scenario represented by the PD lagoons requires research aimed at the preservation of transitional littoral sites, an almost unique investigation theatre. Our results contribute to the identification of the causes of the current decline in the production of mussels in the SC, providing the implementation of an experimental step (in situ mussel filtration rates) essential in the planning and management of interventions for enhancing mussel production without neglecting aspects of environmental protection of coastal systems.

## Materials and methods

### Study area

The SC is the largest lagoon of the PD and extends over an area of approximately 30 km^2^ between the two branches of the Po River, namely, the Po di Tolle and the Po di Gnocca (Fig. 1). In the last century, the SC has experienced the greatest morphological variations in the entire PD, mainly due to subsidence with deepening of the inner sector of the basin, salt marsh erosion, sand bar retreat, and a reduction in the lagoon area^55,56^. The lagoon is protected from the sea by a thin strip of land and sandy banks and connects with the Adriatic Sea through two mouths. The northern sector is characterised by depths ranging between ~ 2 and ~ 2.5 m, the central sector by depths between ~ 1.4 and ~ 1.8 m, and the southern sector presents two dredging canals ~ 3 m deep. However, it represents the shallower (< 1 m) area of the lagoon, especially its western side, characterised by a series of old submerged embankments (Fig. 1c). Water circulation within the SC mainly depends on tidal currents and is stronger at the height of the two mouths and tends to decrease towards the northern part of the SC^9^.

The freshwater inputs from the Po River branches significantly affect the salinity and transport of organic and inorganic materials in the SC, with a minor role played by two dewatering pumps (Bonello—6500 l/s—and Paltanara) at the central-western edge^22^. Together with subsidence, the increase in salinity in the last century led to the disappearance of freshwater rudd fishes (Scardole, in Italy, from which the name “Sacca degli Scardovari” is derived). Temperature shows high temporal and spatial variability depending on the season and tidal phase^15,22^. The SC is the most productive shellfish site for PD, with the production of mussels, clams, and oysters involving approximately 1500 workers. The morphological features of SC generate different environments, such that the shallower central and southern sectors represent farming sites for bivalve molluscs, and the northern sector hosts mussel and oyster farming facilities.

Under the present climate, shellfish production in the area has experienced a crisis (Fig. S4), recently amplified by the massive growth of the invasive blue crab (*C. sapidus*), which has led to the collapse of the entire economically productive system.

### Experimental plan

Sampling stations were placed along two N‒S and E‒W directions to address the influence of the Po River and dewatering pumps on the system (Fig. 1d, Table 2). At each station, the temperature, salinity, and fluorescence were measured using a CTD SBE 19 plus. Discrete water samples were collected using a Niskin bottle deployed horizontally to collect water from the surface (0 m) and bottom (20 cm from the bottom) layers for the analyses of total phytoplankton biomass and related size classes, chemotaxonomy and nutrient concentrations. At stations < 1 m in depth, water samples were collected only from the surface (0 m). Sampling activities were carried out from a boat made available by the ‘Consorzio Pescatori del Polesine di Scardovari’ between April 2022 and May 2023. The sampling time for each cruise was less than two hours to reduce the impact of tidal phases on the considered variables (Fig. S5). To evaluate phytoplankton community variations at 24 h intervals, in the presence of the Po River minimum flow rate and high tidal phases, two samplings were carried out on July 15–16, 2022, in addition to an in situ mesocosm experiment in which the filtering activity of 400 ± 30 mussels was evaluated in a confined water volume of 600 l using a polyethylene transparent chamber allowing light penetration.

**Table 2:** Geographical coordinates of the sampled stations with relative depths and sectors.

Sector	Station	Latitude (degrees north)	Longitude (degrees east)	Bottom depth (m)
1	1	44.893084	12.422660	2.2
1	2	44.880483	12.412467	2.6
1	3	44.880266	12.433924	2.9
2	4	44.867000	12.397683	2.2
2	5	44.865671	12.415762	2.1
2	6	44.860862	12.434021	2.1
2	7	44.851037	12.415617	1.2
3	8	44.843967	12.412433	1.4
3	9	44.844217	12.437383	2.6
3	10	44.822467	12.416507	0.78
3	11	44.822075	12.424717	~ 3*

Sampling date: April 22, 2022 [April 2022], July 15, 2022 [July (1) 2022], July 16, 2022 [July (2) 2022], January 18, 2023 [January 2023], May 25, 2023 [May 2023].

*Bottom depth strongly subjected to changes due to particulate river transport.

*Nutrient concentration.* To determine the nutrient concentrations (N-NO_3_, N-NH_3_, Si-SiO_4_, P-PO_4_), water samples were collected directly from the Niskin bottles by means of a syringe equipped with filters (0.45 μm polycarbonate Whatman) and stored at − 20 °C in 20 ml low-density polyethylene (LDPE) containers until laboratory analysis. The analyses were carried out using a Systea (SYSTEA Analytical Technologies S.p.A.—Anagni (FR) Italy) Easy Chem Plus analyser according to the methodology of^57^.

#### Total phytoplankton biomass and size classes

For the analysis of total phytoplankton biomass, 100 ml of lagoon water was filtered through a GF/F filter (total Chl a) (Whatman, Maidstone, 143 United Kingdom, 25 mm diameter, 0.7 µm) and then immediately cryopreserved in liquid nitrogen within cryovials.

For the determination of the size classes of the microfraction (200–20 μm size cells), nanofraction (20–2 μm size cells), and picofraction (< 2 μm), a series of serial filtrations were carried out using different meshes, allowing for the selection of different subclasses of particles. In particular, 100 ml of lagoon water was prefiltered by a 20 μm screen to let only phytoplankton < 20 μm pass through, and the phytoplankton were then filtered through a 0.7 µm GF/F filter (Whatman, Maidstone, United Kingdom, 25 mm diameter), which therefore only had a biomass between 0.7 and 20 μm (Chl a < 20 μm). A second screen with 2 μm pores was used for the filtration of 100 ml of lagoon water to pass only phytoplankton < 2 μm, and then the volume was filtered through a 0.7 µm GF/F filter (Whatman, Maidstone, United Kingdom, 25 mm diameter), which therefore only had biomass between 0.7 and 2 μm (Chl a < 2 μm). All GF/F filters (Whatman, Maidstone, United Kingdom, 25 mm diameter) were immediately stored in liquid nitrogen after filtration until laboratory analyses. The contributions of total phytoplankton biomass (total Chl a) and the micro-, nano-, and picosize classes were evaluated through analyses of Chl a and phaeopigment (Phaeo) levels according to^58^ using a Shimadzu spectrofluorometer (Shimadzu 175 Corporation, Maryland, United States; model Spex Fluoromax). The contribution of each size class was derived as follows: microsize = (total Chl a – Chl a < 20 μm); nanosize = (Chl a < 20 μm—Chl a < 2 μm); picosize = (Chl a < 2 μm). Each size fraction was calculated as a percentage of the total biomass.

#### Chemiofunctional groups

The phytoplankton composition was estimated by analysing photosynthetic pigments using HPLC. This technique has been helpful for studying phytoplankton communities in aquatic ecosystems^59,60^. HPLC pigment separation was performed on an Agilent 1100 HPLC system (Agilent Technologies, Santa Clara, CA, USA) equipped with an HP 1050 photodiode array detector and an HP 1046A fluorescence detector for the determination of chlorophyll degradation products, according to the methods outlined in^61^. Instrument calibration was performed using external standard pigments provided by the International Agency for 14C Determination-VKI Water Quality Institute. Pigment concentrations were used to estimate the contributions of the main chemofunctional groups to total the Chl a concentration using the CHEMTAX 1.95 matrix factorisation programme following^59,62^. The considered pigments are chlorophyll c_2_ (Chl c_2_), Mg-2,4-divinyl pheoporphyrin (MGDVP), peridinin (Perid), prasinoxanthin (Prasino), fucoxanthin (Fuco), 19'-hexanoyloxyfucoxanthin (19'HF), violaxanthin (Viola), diadinoxanthin (Diadino), anteraxanthin (Anterax), alloxanthin (Allox), diatoxanthin (Diato), zeaxanthin (Zeax), lutein (Lut), chlorophyll b (Chl b), β-carotene (beCar), and Chl a. Due to the high environmental variability of the study area, five different matrices were generated based on the available literature and following the methodology indicated by^59^. The initial and final ratio matrix used for the estimation of chemotaxonomically defined groups is reported in Table S3.

#### Evaluation of in situ mussel filtration activity

To investigate the influence of filter feeders on food web flow, an in situ experiment on the filtration activity of mussels was conducted near station 10 on July 16, 2022. To this end, three transparent polyethylene chambers (600 l volume) were built to allow light to pass through, placed close to the bottom, and raised to entrap the above water column. A 1.5 m rope with 400 ± 30 mussels attached was placed within each chamber and monitored for 12 h. Sampling was performed every 6 h (T0: start, T6: after 6 h; T12: after 2 h) to determine the total phytoplankton biomass (Chl a) and the size classes of micro-, nano-, and pico-phytoplankton within and outside the chamber (control). To avoid the resuspension of particulate matter inside the chamber, a manual peristaltic pump was used to collect water samples from the surface (0 m), intermediate (1 m), and bottom (2 m) depths. The average biomass filtered by mussels was 7.5 ± 0.60 kg on average in each chamber.

#### Statistical analyses

Statistical analyses were carried out using PAST Palaeontological Statistics Version 4.15 (Øyvind Hamme) and GraphPad Prism 10.2.3 (GraphPad Software, Inc., San Diego, CA). Two-way PERMANOVA was performed to evaluate the extent of statistically significant differences between sectors (northern, central, and southern) and seasonal variability within the SC. Sts 1, 2, and 3 were grouped into sector 1; Sts 4, 5, 6, and 7 were grouped into sector 2; and Sts 8, 9, 10, and 11 were grouped into sector 3. The Bray‒Curtis index was used as a similarity index. Significance was computed by permuting the group membership, with 9999 replicates^63^. To show the degree of similarity between stations, network plot analysis was conducted based on the Bray‒Curtis similarity index, in which the degree of similarity between different stations was established based on the chemofunctional composition of the communities in the surface (blue dots) and bottom (red dots) layers. A 50% cut-off value was used to show only edges between nodes that were more than 50% similar. The nodes were arranged based on the Fruchterman-Reingold algorithm^64^, and stations with a similar composition were connected with a thick black line. For the analyses of total phytoplankton biomass and community structure in the mussel filtering mesocosm experiments, two-way ANOVA was carried out.

NMDS based on a distance matrix computed with the Bray‒Curtis dissimilarity index war was performed on the overall dataset^65^. The environmental variables (temperature, salinity, N-NO_3_, N-NH_3_, Si-SiO_4_, P-PO_4_ and the N/P ratio) were not included in the ordination, and the correlation coefficients between each environmental variable and the NMDS scores are presented as vectors from the origin.

### Ethical approval

All authors have agreed to be listed and approved the submitted version of the manuscript, disclosing the absence of any potential source of conflict of interest, in accordance with your journal’s policy.

## Supplementary Information

Supplementary Legends.

Supplementary Figure 1.

Supplementary Figure 2.

Supplementary Figure 3.

Supplementary Figure 4.

Supplementary Figure 5.

Supplementary Table 1.

Supplementary Table 2.

Supplementary Table 3.

Supplementary Table 4.

## Data Availability

The datasets used and/or analysed during the current study are available from the corresponding author upon reasonable request.

## References

[1] Giosan, L., Syvitski, J., Constantinescu, S. & Day, J. Climate change: Protect the world’s deltas. *Nature* **516**, 31–33 (2014). 25471866 10.1038/516031a

[2] Pérez-Ruzafa, A., Pérez-Ruzafa, I. M., Newton, A. & Marcos, C. Coastal Lagoons: Environmental Variability, Ecosystem Complexity, and Goods and Services Uniformity. In *Coasts and Estuaries* 253–276 (Elsevier, 2019). 10.1016/B978-0-12-814003-1.00015-0.

[3] Pérez-Ruzafa, A., Pérez-Marcos, M. & Marcos, C. Coastal lagoons in focus: Their environmental and socioeconomic importance. *J. Nat. Conserv.* **57**, 125886 (2020).

[4] Eslami, S. *et al.* Projections of salt intrusion in a mega-delta under climatic and anthropogenic stressors. *Commun. Earth Environ.* **2**, 142 (2021).

[5] Aurelle, D. *et al.* Biodiversity, climate change, and adaptation in the Mediterranean. *Ecosphere* **13**, e3915 (2022).

[6] Valle-Levinson, A. *Contemporary Issues in Estuarine Physics* (Cambridge University Press, 2010).

[7] Brando, V. E. *et al.* High-resolution satellite turbidity and sea surface temperature observations of river plume interactions during a significant flood event. *Ocean Sci.* **11**, 909–920 (2015).

[8] Horner-Devine, A. R., Hetland, R. D. & MacDonald, D. G. Mixing and transport in coastal river plumes. *Annu. Rev. Fluid Mech.* **47**, 569–594 (2015).

[9] Maicu, F., Ferrarin, C., De Pascalis, F. & Umgiesser, G. Hydrodynamics of the Po river-delta-sea system. *J. Geophys. Res. Oceans* **123**, 6349 (2018).

[10] Bellafiore, D. *et al.* Coastal mixing in multiple-mouth deltas: A case study in the Po delta, Italy. *Estuar. Coast. Shelf Sci.* **226**, 106254 (2019).

[11] Nicholls, R. J. *et al.* Sustainable deltas in the anthropocene. In *Deltas in the Anthropocene* (eds Nicholls, R. J. *et al.*) 247–279 (Springer, 2020).

[12] Bellafiore, D. *et al.* Saltwater intrusion in a Mediterranean delta under a changing climate. *J. Geophys. Res. Oceans* **126**, e2020JC016437 (2021).

[13] Collados-Lara, A.-J., Gómez-Gómez, J.-D., Pulido-Velazquez, D. & Pardo-Igúzquiza, E. An approach to identify the best climate models for the assessment of climate change impacts on meteorological and hydrological droughts. *Nat. Hazards Earth Syst. Sci.* **22**, 599–616 (2022).

[14] Ninfo, A., Ciavola, P. & Billi, P. The Po delta is restarting progradation: geomorphological evolution based on a 47-years earth observation dataset. *Sci. Rep.* **8**, 3457 (2018). 29472570 10.1038/s41598-018-21928-3 PMC5823860

[15] Andreoli, C. *et al.* Phytoplankton and chemicophysical parameters of the Scardovari Lagoon (Po Delta, North Adriatic Sea) during 1991 and 1992. *Giorn. Bot. Ital.* **128**, 1007–1027 (1994).

[16] Braga, F. *et al.* Mapping turbidity patterns in the Po river prodelta using multi-temporal Landsat 8 imagery. *Estuar. Coast. Shelf Sci.* **198**, 555–567 (2017).

[17] Colombani, N., Giambastiani, B. M. S. & Mastrocicco, M. Impact of climate variability on the salinization of the coastal wetland-aquifer system of the Po Delta, Italy. *J. Water Supply Res. Technol. AQUA*10.2166/aqua.2017.115 (2017).

[18] Sfriso, A., Facca, C., Bon, D., Giovannone, F. & Buosi, A. Using phytoplankton and macrophytes to assess the trophic and ecological status of some Italian transitional systems. *Cont. Shelf Res.* **81**, 88–98 (2014).

[19] Surian, N. & Rinaldi, M. Morphological response to river engineering and management in alluvial channels in Italy. *Geomorphology* **50**, 307–326 (2003).

[20] Surian, N., Ziliani, L., Comiti, F., Lenzi, M. A. & Mao, L. Channel adjustments and alteration of sediment fluxes in gravel-bed rivers of North-Eastern Italy: Potentials and limitations for channel recovery. *River Res. Appl.* **25**, 551–567 (2009).

[21] Rinaldi, M., Surian, N., Pellegrini, L., Maraga, F. & Turitto, O. Attuali conoscenze sull’evoluzione recente di corsi d’acqua del Bacino Padano ed implicazioni per la gestione e riqualificazione fluviale. *Biol. Ambient.* **24**, 29–40 (2010).

[22] D’Alpaos, L. Hydrodynamic circulation and the morphological evolution of the Sacca di Scardovari: The po delta lagoons (eds Consorzio di Bonifica Delta del Po & Taglio di Po). 3–80 (Regione del Veneto, 2014).

[23] Antonellini, M., Allen, D., Mollema, P., Capo, D. & Greggio, N. Groundwater freshening following coastal progradation and land reclamation of the Po Plain, Italy. *Hydrogeol. J.* **23**, 1009–1026 (2015).

[24] Da Lio, C., Carol, E., Kruse, E., Teatini, P. & Tosi, L. Saltwater contamination in the managed low-lying farmland of the Venice coast, Italy: An assessment of vulnerability. *Sci. Total Environ.* **533**, 356–369 (2015). 26172603 10.1016/j.scitotenv.2015.07.013

[25] Taramelli, A. *et al.* Assessing Po river deltaic vulnerability using earth observation and a Bayesian belief network model. *Water* **12**, 2830 (2020).

[26] Corbau, C., Simeoni, U., Zoccarato, C., Mantovani, G. & Teatini, P. Coupling land use evolution and subsidence in the Po Delta, Italy: Revising the past occurrence and prospecting the future management challenges. *Sci. Total Environ.* **654**, 1196–1208 (2019). 30841394 10.1016/j.scitotenv.2018.11.104

[27] Gervasio, M. P., Soana, E., Vincenzi, F., Magri, M. & Castaldelli, G. Drought-induced salinity intrusion affects nitrogen removal in a deltaic ecosystem (Po River Delta, Northern Italy). *Water* **15**, 2405 (2023).

[28] Ibáñez, C. Impacts of climate change on Mediterranean coastal wetlands and lagoons. In *Impacts of Climate Change in the Coastal Zone* (ed. Yáñez-Arancibia, A.) 127–142 (Instituto De Ecología A.C. INECOL, Instituto Nacional De Ecología INE-SEMARNAT, 2010).

[29] Tamburini, E., Turolla, E., Fano, E. A. & Castaldelli, G. Sustainability of mussel (*Mytilus galloprovincialis*) farming in the Po river delta, Northern Italy, based on a life cycle assessment approach. *Sustainability* **12**, 3814 (2020).

[30] Montanari, A. *et al.* Why the 2022 Po River drought is the worst in the past two centuries. *Sci. Adv.* **9**, eadg8304 (2023). 37556532 10.1126/sciadv.adg8304 PMC10411875

[31] Sacchi, C. F. & Renzoni, A. L’ècologie de *Mytilus galloprovincialis* (Lam.) dans l’ètang litoral du Fusaro et les rythmes saisonies et nychthèmèraux des facteurs environnants. *Publ. Staz. Zool. Napoli* **32**, 255–293 (1962).

[32] Navarro, E., Iglesias, J. I. P., Camacho, A. P. & Labarta, U. The effect of diets of phytoplankton and suspended bottom material on feeding and absorption of raft mussels (*Mytilus galloprovincialis* Lmk). *J. Exp. Mar. Biol. Ecol.* **198**, 175–189 (1996).

[33] Babarro, J. M. F., Fernández-Reiriz, M. J. & Labarta, U. *In situ* absorption efficiency processes for the cultured mussel *Mytilus galloprovincialis* in Ría de Arousa (north-west Spain). *J. Mar. Biol. Assoc. U. K.* **83**, 1059–1064 (2003).

[34] Maar, M., Nielsen, T. & Petersen, J. Depletion of plankton in a raft culture of *Mytilus galloprovincialis* in Ría de Vigo, NW Spain. II. *Zooplankton Aquat. Biol.* **4**, 127–141 (2008).

[35] Dame, R. F. & Prins, T. C. Bivalve carrying capacity in coastal ecosystems. *Aquat. Ecol.* **31**, 409–421 (1998).

[36] Nakamura, Y. & Kerciku, F. Effects of filter-feeding bivalves on the distribution of water quality and nutrient cycling in a eutrophic coastal lagoon. *J. Mar. Syst.* **26**, 209–221 (2000).

[37] Filgueira, R., Grant, J., Bacher, C. & Carreau, M. A physical–biogeochemical coupling scheme for modeling marine coastal ecosystems. *Ecol. Inform.* **7**, 71–80 (2012).

[38] Salmaso, N., Naselli-Flores, L. & Padisák, J. Functional classifications and their application in phytoplankton ecology. *Freshw. Biol.* **60**, 603–619 (2015).

[39] Colombo, G. Results of hydrobiological investigations on a brackish water bay (Sacca degli Scardovari) of the Po river delta. *Rapp. Comm. Int. Mer. Médit.* **27**, 89–92 (1981).

[40] Litchman, E., Edwards, K., Klausmeier, C. & Thomas, M. Phytoplankton niches, traits and eco-evolutionary responses to global environmental change. *Mar. Ecol. Prog. Ser.* **470**, 235–248 (2012).

[41] Naselli-Flores, L. & Padisák, J. Ecosystem services provided by marine and freshwater phytoplankton. *Hydrobiologia* **850**, 2691–2706 (2023). 35106010 10.1007/s10750-022-04795-y PMC8795964

[42] Spillman, C. M., Imberger, J., Hamilton, D. P., Hipsey, M. R. & Romero, J. R. Modelling the effects of Po River discharge, internal nutrient cycling and hydrodynamics on biogeochemistry of the Northern Adriatic Sea. *J. Mar. Syst.* **68**, 167–200 (2007).

[43] Mangoni, O. *et al.* Structure and photosynthetic properties of phytoplankton assemblages in a highly dynamic system, the Northern Adriatic Sea. *Estuar. Coast. Shelf Sci.* **77**, 633–644 (2008).

[44] Gallardi, D. *et al.* Effects of extended ambient live holding on cultured blue mussels (*Mytilus edulis* L.) with reference to condition index, lipid profile, glycogen content and organoleptic testing. *Aquaculture* **430**, 149–158 (2014).

[45] van der Schatte Olivier, A. *et al.* A global review of the ecosystem services provided by bivalve aquaculture. *Rev. Aquac.* **12**, 3–25 (2020).

[46] Moore, D., Heilweck, M. & Petros, P. Cultivate shellfish to remediate the atmosphere. In *Aquaculture: Ocean Blue Carbon Meets UN-SDGS* (ed. Moore, D.) 35–63 (Springer, 2022). 10.1007/978-3-030-94846-7_2.

[47] Simeoni, U. & Corbau, C. A review of the Delta Po evolution (Italy) related to climatic changes and human impacts. *Geomorphology* **107**, 64–71 (2009).

[48] Leffler, C. Some effects of temperature on the growth and metabolic rate of juvenile blue crabs, callinectes sapidus, in the laboratory. *Mar. Biol.* **14**, 104–110 (1972).

[49] Azra, M., Chen, J.-C. & Abol-Munafi, A. Thermal tolerance and locomotor activity of blue swimmer crab portunus pelagicus instar reared at different temperatures. *J. Therm. Biol.* **74**, 234–240 (2018). 29801633 10.1016/j.jtherbio.2018.04.002

[50] Molina, A., Cerrato, R. & Nye, J. Population level differences in overwintering survivorship of blue crabs (*Callinectes sapidus*): A caution on extrapolating climate sensitivities along latitudinal gradients. *PLoS ONE* **16**, e0257569 (2021). 34547045 10.1371/journal.pone.0257569 PMC8454986

[51] Marchessaux, G. *et al.* The invasive blue crab *Callinectes sapidus* thermal response: Predicting metabolic suitability maps under future warming Mediterranean scenarios. *Front. Mar. Sci.* **9**, 1055404 (2022).

[52] Junda, L. Predator–prey interactions between blue crabs and ribbed mussels living in clumps. *Estuar. Coast. Shelf Sci.* **32**, 61–69 (1991).

[53] Prado, P. *et al.* Trophic role and predatory interactions between the blue crab, *Callinectes sapidus*, and native species in open waters of the Ebro Delta. *Estuar. Coast. Shelf Sci.* **298**, 108638 (2024).

[54] Ortega-Jiménez, E., Cuesta, J., Laiz, I. & González-Ortegón, E. Diet of the Invasive Atlantic Blue Crab *Callinectes sapidus* Rathbun, 1896 (Decapoda, Portunidae) in the Guadalquivir Estuary (Spain). *Estuaries Coasts* **47**, 1075–1085 (2024).

[55] Mattichio, B. Evoluzione morfologica recente della Sacca degli Scardovari. 16–55 (2009).

[56] Parrinello, G., Bizzi, S. & Surian, N. The retreat of the delta: A geomorphological history of the Po river basin during the twentieth century. *Water Hist.* **13**, 117–136. 10.1007/s12685-021-00279-3 (2021).

[57] Grasshoff, K., Ehrhardt, M. & Kremling, K. *Automated Chemical Analysis.* (Grasshoff, K., Ehrardt, M., Kremlin, K., New York, 1983).

[58] Holm-Hansen, O., Lorenzen, C. J., Holmes, R. W. & Strickland, J. D. H. Fluorometric determination of chlorophyll. *ICES J. Mar. Sci.* **30**, 3–15. 10.1093/icesjms/30.1.3 (1965).

[59] Latasa, M. Improving estimations of phytoplankton class abundances using CHEMTAX. *Mar. Ecol. Prog. Ser.* **329**, 13–21 (2007).

[60] Roy, S. *et al.* (eds) *Phytoplankton Pigments: Characterization, Chemotaxonomy and Applications in Oceanography* (Cambridge University Press, 2011). 10.1017/CBO9780511732263.

[61] Vidussi, F., Claustre, H., Bustillos-Guzmàn, J., Cailliau, C. & Marty, J.-C. Determination of chlorophylls and carotenoids of marine phytoplankton: Separation of chlorophyll *a* from divinylchlorophyll *a* and zeaxanthin from lutein. *J. Plankton Res.* **18**, 2377–2382 (1996).

[62] Mackey, M., Mackey, D., Higgins, H. & Wright, S. CHEMTAX—A program for estimating class abundances from chemical markers: Application to HPLC measurements of phytoplankton. *Mar. Ecol. Prog. Ser.* **144**, 265–283 (1996).

[63] Anderson, M. J. A new method for non-parametric multivariate analysis of variance. *Austral Ecol.* **26**, 32–46 (2001).

[64] Fruchterman, T. M. J. & Reingold, E. M. Graph drawing by force-directed placement. *Softw. Pract. Exp.* **21**, 1129–1164 (1991).

[65] Taguchi, Y.-H. & Oono, Y. Relational patterns of gene expression via non-metric multidimensional scaling analysis. *Bioinformatics* **21**, 730–740 (2005). 15509613 10.1093/bioinformatics/bti067

