# Author Correction: Phytoplankton dynamics in a shellfish farming lagoon in a deltaic system threatened by ongoing climate change

**DOI:** 10.1038/s41598-024-76161-y

**Published:** 2024-10-22

**Authors:** Francesco Bolinesi, Emanuele Rossetti, Olga Mangoni

**Affiliations:** 1 Dipartimento di Biologia, Università degli Studi di Napoli Federico II, Complesso di Monte Sant’Angelo, Via Cinthia 21, 80126 Napoli, Italy; 2 CoNISMa, Piazzale Flaminio 9, 00196 Roma, Italy; 3 Consorzio Cooperative Pescatori del Polesine O.P. S.C.Ar.L., 45018 Scardovari, Rovigo, Italy

Correction to: *Scientific Reports *10.1038/s41598-024-70492-6, published online 21 August 2024

The original version of this Article contained errors in Figure 3, where color scale bars with the concentration of Chlorophyll a (μg/l Chl a) were omitted. The original Figure 3 and accompanying legend appear below.

**Fig. 3:**
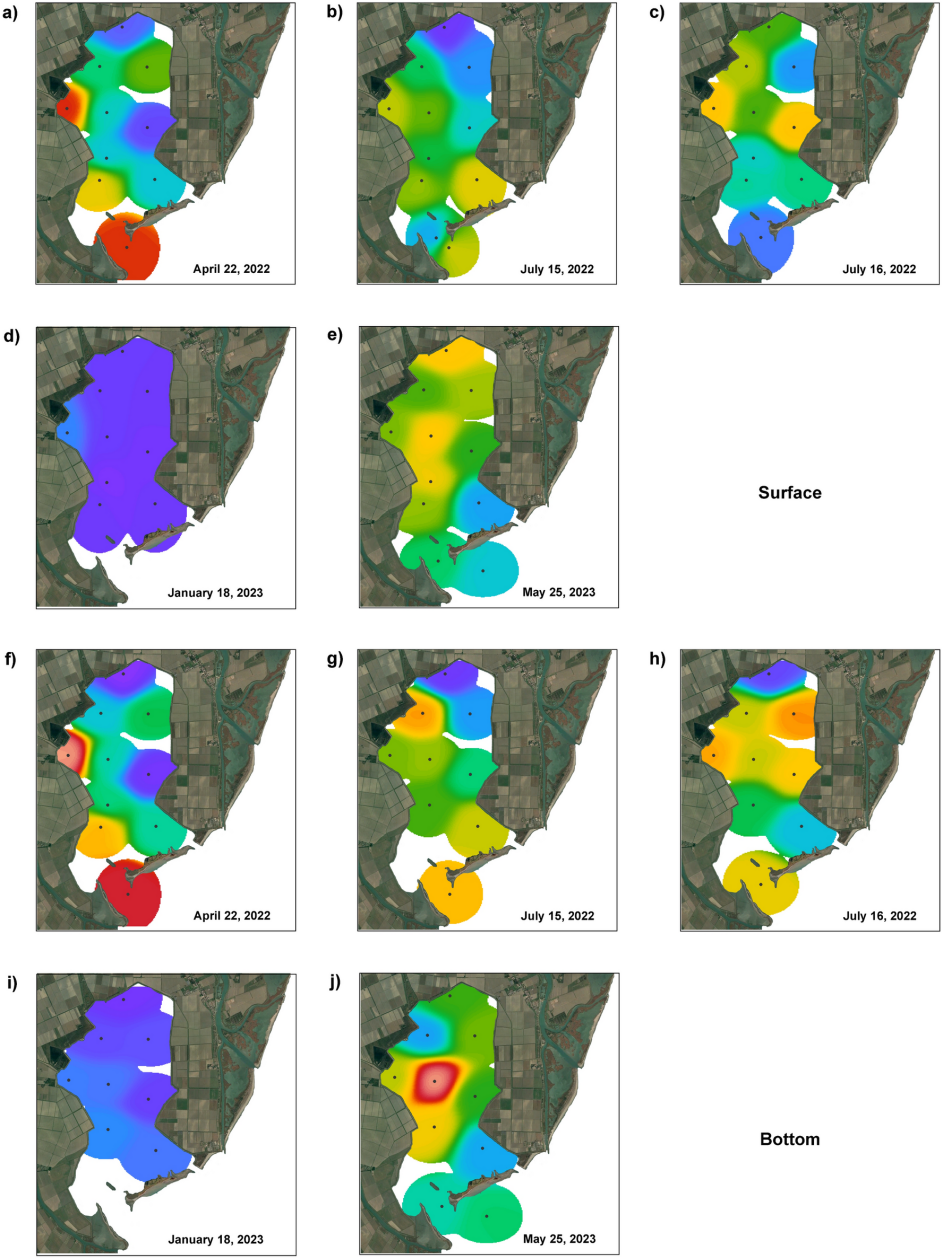
Spatial and temporal variation in total phytoplankton biomass (Chl a, μg/l) in the surface and bottom layers for each sampling period. 26 April 2022: (**a**) surface and (**f**) bottom; 15 July (1) 2022: (**b**) surface and (**g**) bottom; 16 July (2) 2022: (**c**) surface and (**h**) bottom; January 18, 2023: (**d**) surface and (**i**) bottom; 25 May 2023: (**e**) surface and (**j**) bottom. Map data: Google, CNES/Airbus. Software used: Google Earth Pro and Adobe Photoshop. The interpolation of values was performed according to weighted-average gridding by Ocean Data View (odv.5.7.1—http://odv.awi.de).

Additionally, Figure S1 from in the Supplementary Information stated incorrect sampling dates from the Po River flooding. The original Figure S1 and accompanying legend appear below.

**Fig. S1:**
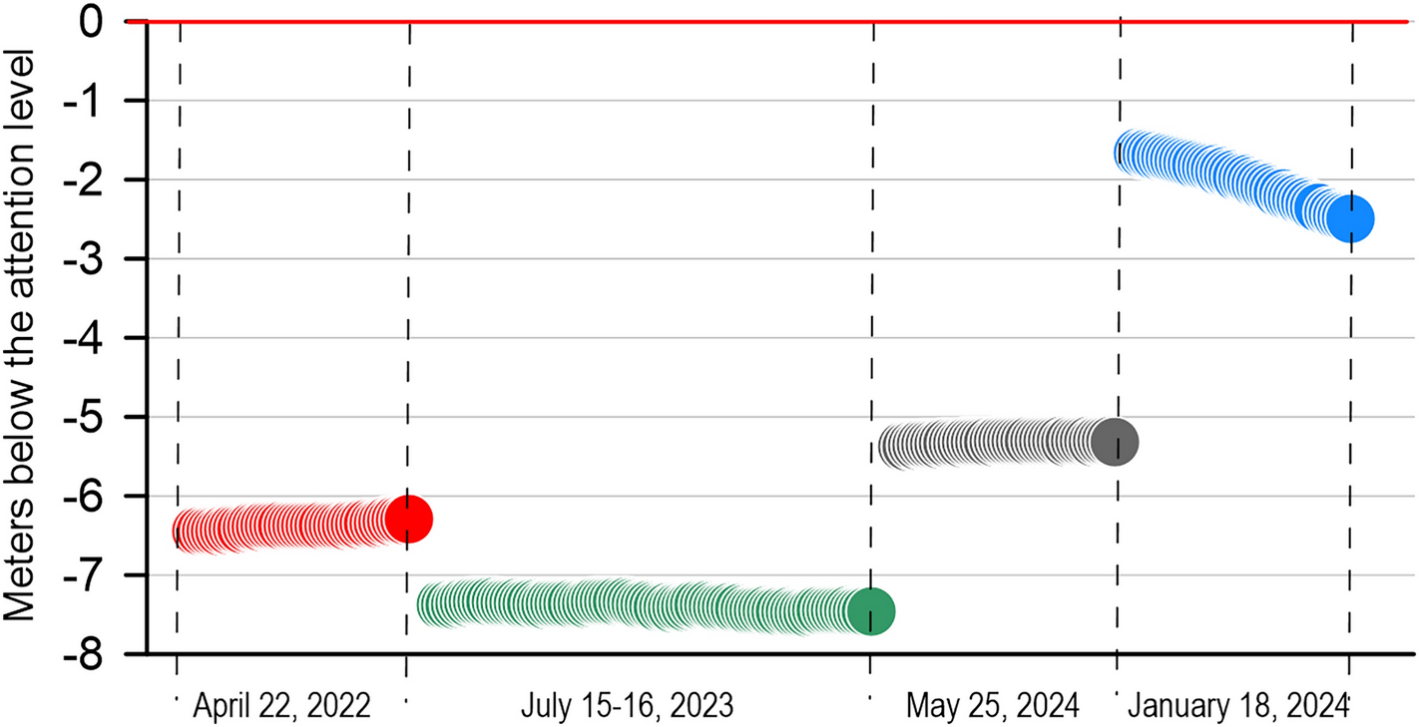
Hydrometric level of the Po River over 24 h for each sampling date. Values are reported as meters below the attention level (0).

The original Article and accompanying Supplementary Information file have been corrected.

