# Supplementary material for: Phytoplankton dynamics in a shellfish farming lagoon in a deltaic system threatened by ongoing climate change
Source: Sci Rep. 2024 Aug 21;14:19424. doi: 10.1038/s41598-024-70492-6 (PMC11339385; doi:10.1038/s41598-024-70492-6)
Supplement: Supplementary file 1 — Supplementary Legends. [file 41598_2024_70492_MOESM1_ESM.docx]

### **Supplementary material (figure legends)**

**Figure S1.** Hydrometric level of the Po River over 24h for each sampling date. Values are reported as meters below the attention level (0).

**Figure S2.** Temporal variation in salinity in the surface layer (panel above) and bottom (lower panel) in all sampling periods.

**Figure S3.** Temporal variation in Temperature (°C) in the surface layer (panel above) and bottom (lower panel) in all sampling periods.

**Figure S4.** Annual production of mussels (quintals) in the SC between 2010 and 2023.

**Figure S5.** Tidal range during each sampling period. Blu and red lines represent the maximum and minimum levels for the SC (<https://it.tideschart.com/Italy/Veneto/Provincia-di-Rovigo/Scardovari/>).
