# Supplementary figures and images for: Phytoplankton dynamics in a shellfish farming lagoon in a deltaic system threatened by ongoing climate change
Source: Sci Rep. 2024 Aug 21;14:19424. doi: 10.1038/s41598-024-70492-6 (PMC11339385; doi:10.1038/s41598-024-70492-6)

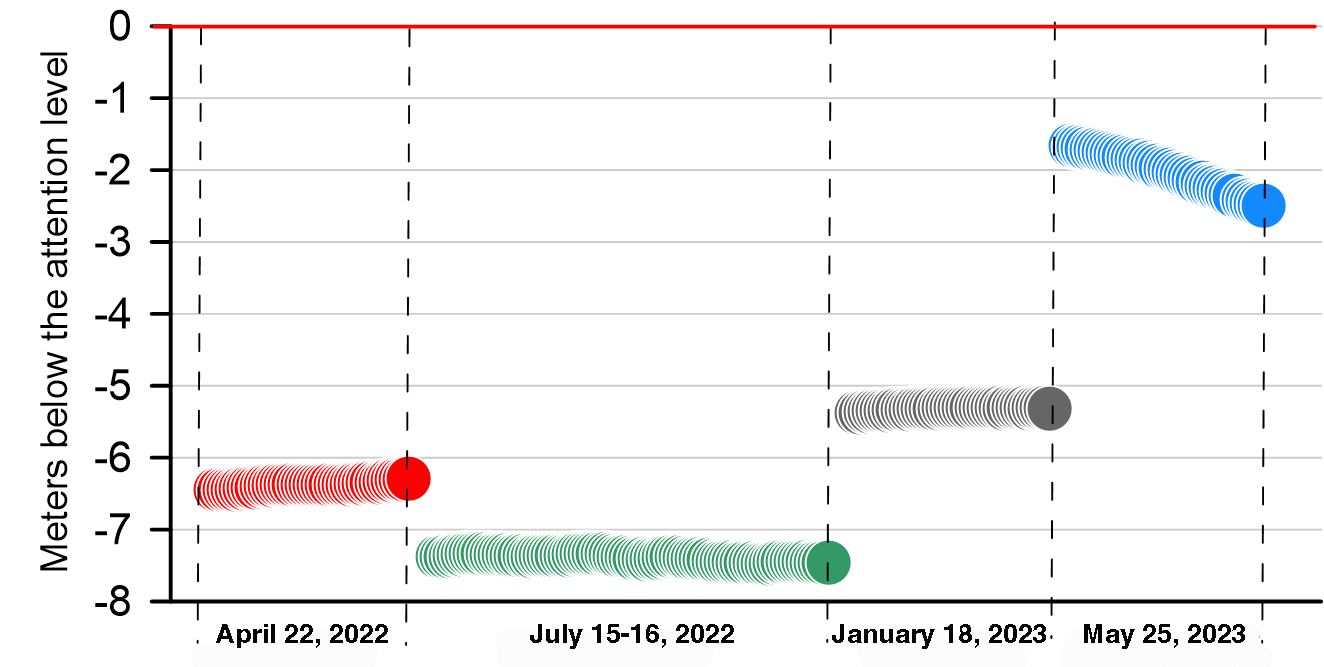

Supplement: Supplementary file 2 — Supplementary Figure 1. [file 41598_2024_70492_MOESM2_ESM.tif]

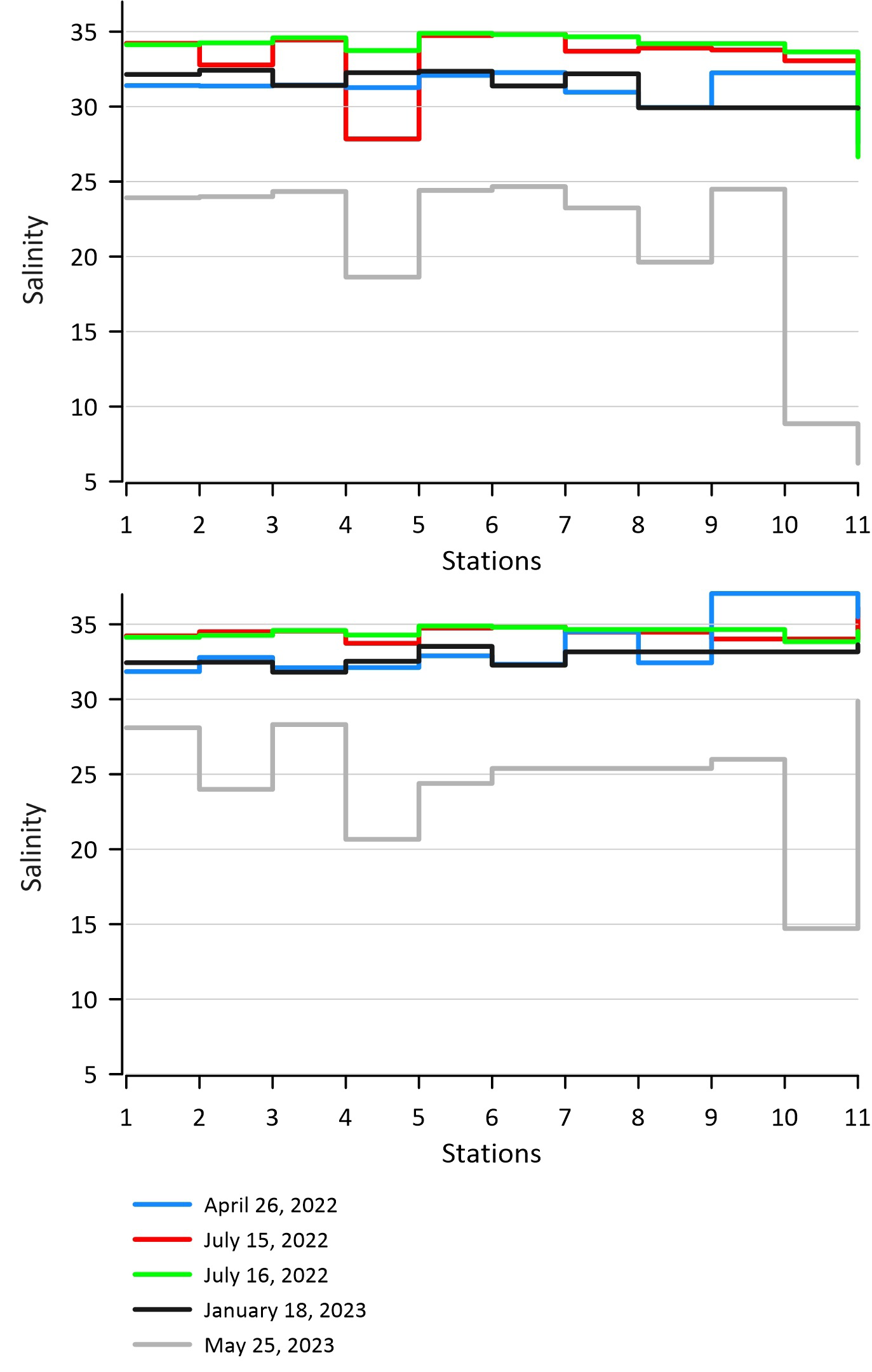

Supplement: Supplementary file 3 — Supplementary Figure 2. [file 41598_2024_70492_MOESM3_ESM.tif]

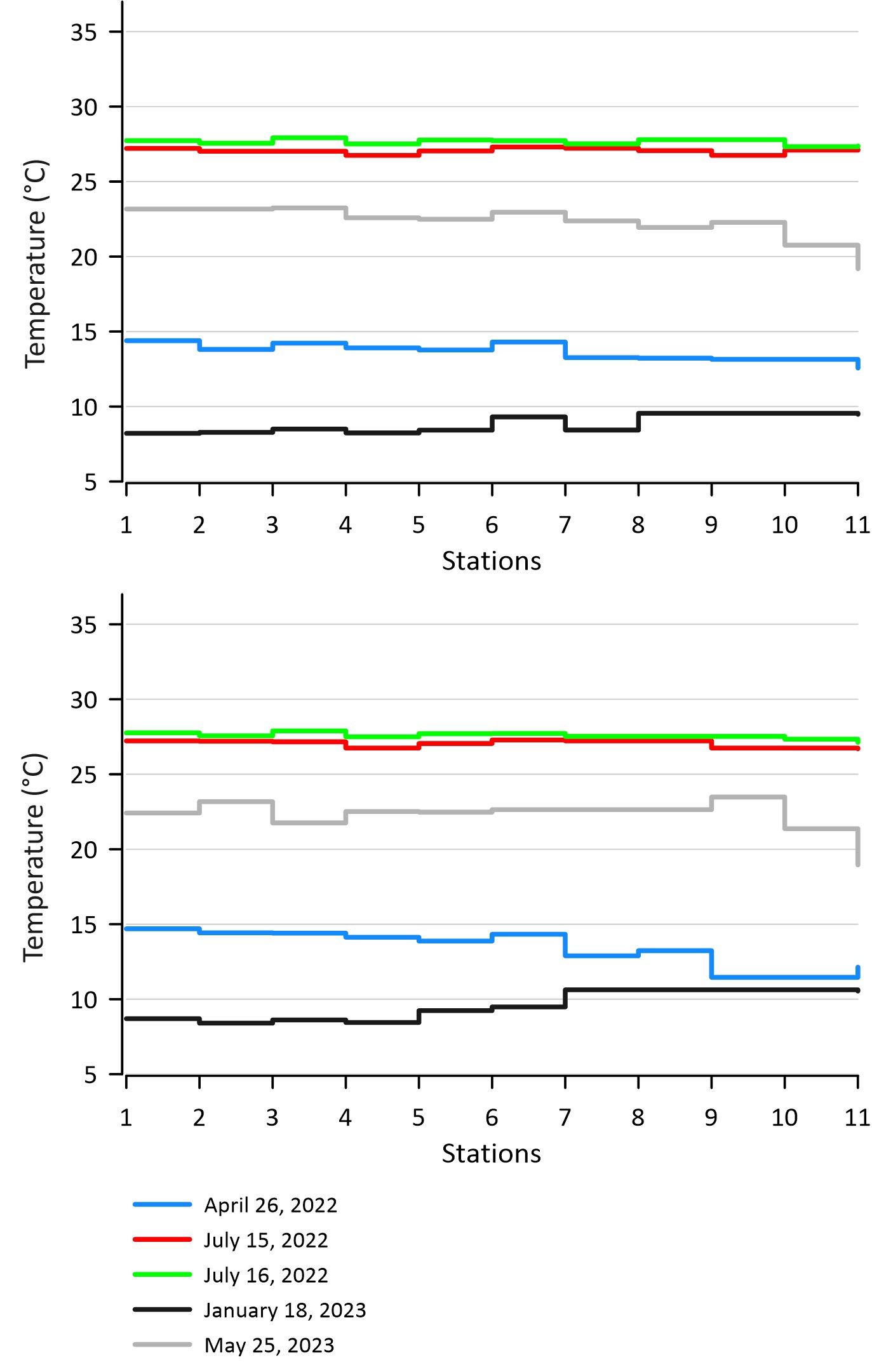

Supplement: Supplementary file 4 — Supplementary Figure 3. [file 41598_2024_70492_MOESM4_ESM.tif]

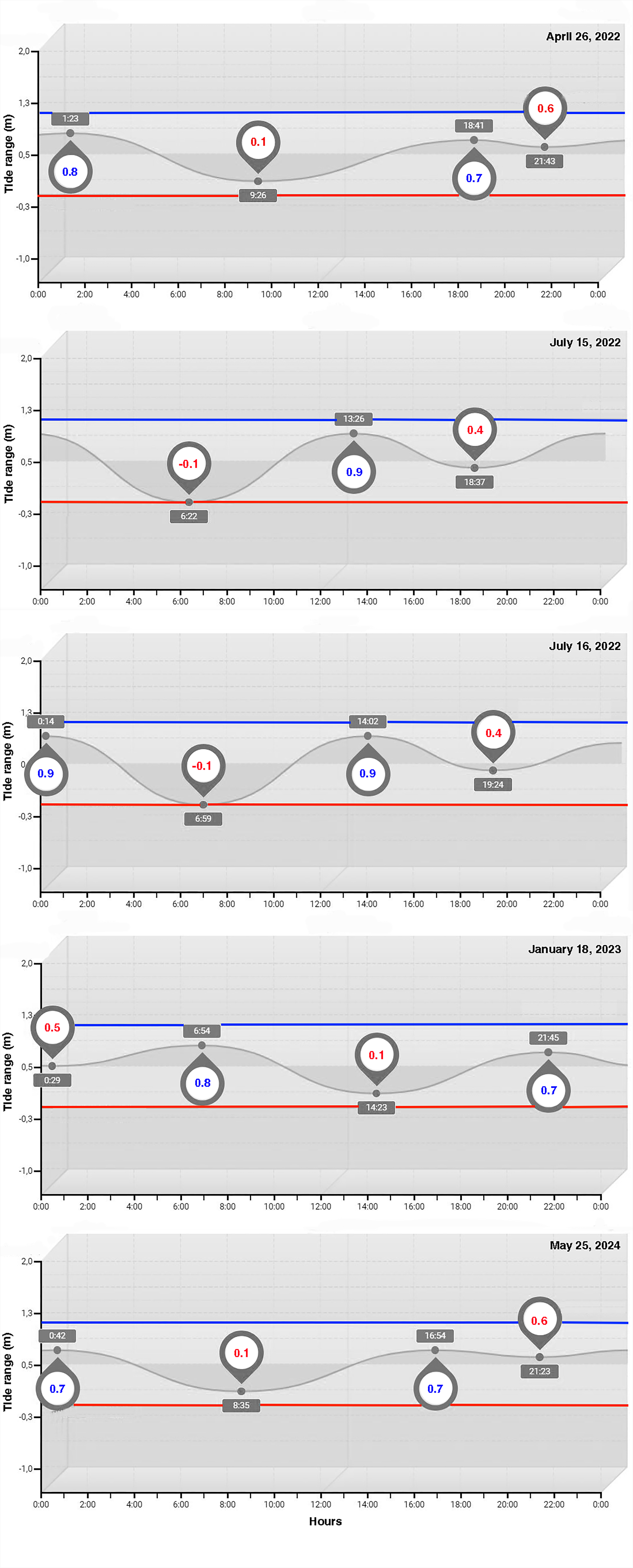

Supplement: Supplementary file 6 — Supplementary Figure 5. [file 41598_2024_70492_MOESM6_ESM.tif]
