# Supplementary material for: Phytoplankton dynamics in a shellfish farming lagoon in a deltaic system threatened by ongoing climate change
Source: Sci Rep. 2024 Aug 21;14:19424. doi: 10.1038/s41598-024-70492-6 (PMC11339385; doi:10.1038/s41598-024-70492-6)
Supplement: Supplementary file 7 — Supplementary Table 1. [file 41598_2024_70492_MOESM7_ESM.docx]

**Table S1**. **Nutrient concentrations (μmol/l) and N:P ratios (N-NO4/P-PO4) at each station sampled in different seasons (months) for the surface (0 m) and bottom layers.**

| **Month** | **Station** | **Layer** | **P-PO4 (μmol/l)** | **N-NO3 (μmol/l)** | **Si-SiO4 (μmol/l)** | **N-NH3 (μmol/l)** | **N-NO4/P-PO4** |
| --- | --- | --- | --- | --- | --- | --- | --- |
| April | 1 | Surface | 0,230 | 1,487 | 1,230 | 5,981 | 6,476 |
| April | 1 | Bottom | 0,274 | 1,105 | 1,277 | 4,711 | 4,027 |
| April | 2 | Surface | 0,213 | 1,640 | 1,325 | 4,632 | 7,700 |
| April | 2 | Bottom | 0,255 | 1,706 | 1,440 | 4,918 | 6,689 |
| April | 3 | Surface | 0,359 | 3,070 | 2,471 | 8,155 | 8,542 |
| April | 3 | Bottom | 0,252 | 1,533 | 1,391 | 8,289 | 6,091 |
| April | 4 | Surface | 0,278 | 1,527 | 1,536 | 6,811 | 5,504 |
| April | 4 | Bottom | 0,141 | 1,605 | 1,661 | 5,290 | 11,406 |
| April | 5 | Surface | 0,245 | 3,491 | 2,376 | 5,796 | 14,261 |
| April | 5 | Bottom | 0,265 | 4,318 | 2,644 | 6,187 | 16,267 |
| April | 6 | Surface | 0,278 | 2,853 | 2,483 | 8,776 | 10,271 |
| April | 6 | Bottom | 0,325 | 2,149 | 2,017 | 8,778 | 6,603 |
| April | 7 | Surface | 0,205 | 3,490 | 1,207 | 8,734 | 17,032 |
| April | 7 | Bottom | 0,263 | 2,745 | 1,137 | 6,713 | 10,438 |
| April | 8 | Surface | 0,253 | 4,170 | 2,148 | 5,103 | 16,513 |
| April | 8 | Bottom | 0,222 | 3,921 | 1,337 | 5,122 | 17,697 |
| April | 9 | Surface | 0,229 | 3,501 | 1,751 | 6,593 | 15,256 |
| April | 9 | Bottom | 0,115 | 2,566 | 1,078 | 4,195 | 22,239 |
| April | 11 | Surface | 0,215 | 5,431 | 2,079 | 10,613 | 25,273 |
| April | 11 | Bottom | 0,165 | 3,029 | 0,941 | 5,585 | 18,360 |
| **Month** | **Station** | **Layer** | **P-PO4 (μmol/l)** | **N-NO3 (μmol/l)** | **Si-SiO4 (μmol/l)** | **N-NH3 (μmol/l)** | **N-NO4/P-PO4** |
| July(1) | 1 | Surface | 0,421 | 0,086 | 3,330 | 2,514 | 0,204 |
| July(1) | 1 | Bottom | 0,430 | 0,580 | 3,423 | 2,429 | 1,350 |
| July(1) | 2 | Surface | 0,505 | 0,022 | 3,954 | 2,865 | 0,044 |
| July(1) | 2 | Bottom | 0,536 | 0,049 | 5,782 | 8,033 | 0,091 |
| July(1) | 3 | Surface | 0,572 | 0,027 | 3,647 | 1,749 | 0,048 |
| July(1) | 3 | Bottom | 0,497 | 0,025 | 3,504 | 7,103 | 0,051 |
| July(1) | 4 | Surface | 0,500 | 0,022 | 2,247 | 3,634 | 0,044 |
| July(1) | 4 | Bottom | 0,492 | 0,027 | 2,787 | 1,754 | 0,054 |
| July(1) | 5 | Surface | 0,504 | 0,440 | 4,329 | 1,335 | 0,873 |
| July(1) | 5 | Bottom | 0,399 | 0,029 | 4,019 | 1,450 | 0,074 |
| July(1) | 6 | Surface | 0,701 | 0,057 | 3,581 | 4,281 | 0,081 |
| July(1) | 6 | Bottom | 0,697 | 0,044 | 4,789 | 5,336 | 0,062 |
| July(1) | 7 | Surface | 0,350 | 0,122 | 3,353 | 4,019 | 0,347 |
| July(1) | 7 | Bottom | 0,327 | 0,022 | 3,552 | 4,055 | 0,066 |
| July(1) | 8 | Surface | 0,430 | 0,086 | 3,138 | 4,009 | 0,201 |
| July(1) | 9 | Surface | 0,129 | 0,163 | 1,070 | 2,251 | 1,263 |
| July(1) | 9 | Bottom | 0,131 | 0,222 | 1,361 | 3,295 | 1,700 |
| July(1) | 10 | Surface | 0,375 | 0,446 | 2,275 | 3,865 | 1,189 |
| July(1) | 11 | Surface | 0,188 | 0,497 | 1,057 | 3,268 | 2,651 |
| July(1) | 11 | Bottom | 0,112 | 0,341 | 1,306 | 5,153 | 3,053 |
| **Month** | **Station** | **Layer** | **P-PO4 (μmol/l)** | **N-NO3 (μmol/l)** | **Si-SiO4 (μmol/l)** | **N-NH3 (μmol/l)** | **N-NO4/P-PO4** |
| July(2) | 1 | Surface | 0,387 | 0,024 | 3,892 | 3,761 | 0,063 |
| July(2) | 1 | Bottom | 0,398 | 0,092 | 3,865 | 4,591 | 0,232 |
| July(2) | 2 | Surface | 0,551 | 0,040 | 6,256 | 1,810 | 0,073 |
| July(2) | 2 | Bottom | 0,524 | 0,043 | 6,620 | 1,810 | 0,083 |
| July(2) | 3 | Surface | 0,554 | 0,061 | 7,904 | 4,200 | 0,109 |
| July(2) | 3 | Bottom | 0,545 | 0,045 | 7,950 | 5,004 | 0,082 |
| July(2) | 4 | Surface | 0,417 | 0,060 | 5,724 | 3,264 | 0,144 |
| July(2) | 4 | Bottom | 0,404 | 0,073 | 4,799 | 3,964 | 0,180 |
| July(2) | 5 | Surface | 0,375 | 0,069 | 5,224 | 1,683 | 0,183 |
| July(2) | 5 | Surface | 0,397 | 0,059 |  | 1,625 | 0,149 |
| July(2) | 6 | Bottom | 0,583 | 0,045 | 7,066 | 3,506 | 0,076 |
| July(2) | 6 | Surface | 0,599 | 0,050 | 6,333 | 3,201 | 0,084 |
| July(2) | 7 | Surface | 0,232 | 0,040 | 3,120 | 2,709 | 0,171 |
| July(2) | 7 | Bottom | 0,226 | 0,043 | 2,545 | 2,039 | 0,191 |
| July(2) | 8 | Surface | 0,371 | 0,053 | 4,370 | 4,632 | 0,143 |
| July(2) | 9 | Surface | 0,322 | 0,048 | 4,110 | 8,911 | 0,150 |
| July(2) | 9 | Bottom | 0,297 | 0,033 | 8,865 |  | 0,111 |
| July(2) | 10 | Surface | 0,460 | 0,028 | 2,440 | 7,803 | 0,061 |
| July(2) | 10 | Bottom | 0,379 | 0,025 | 2,446 | 4,882 | 0,067 |
| **Month** | **Station** | **Layer** | **P-PO4 (μmol/l)** | **N-NO3 (μmol/l)** | **Si-SiO4 (μmol/l)** | **N-NH3 (μmol/l)** | **N-NO4/P-PO4** |
| Jan | 1 | Surface | 0,300 | 2,463 | 2,436 | 0,414 | 8,202 |
| Jan | 1 | Bottom | 0,271 | 2,494 | 2,209 | 1,368 | 9,207 |
| Jan | 2 | Surface | 0,189 | 2,007 | 3,824 | 0,272 | 10,624 |
| Jan | 2 | Bottom | 0,312 | 2,032 | 2,672 | 0,210 | 6,520 |
| Jan | 3 | Surface | 0,247 | 4,254 | 4,016 | 0,538 | 17,246 |
| Jan | 3 | Bottom | 0,336 | 6,045 | 3,870 | 1,075 | 18,006 |
| Jan | 4 | Surface | 0,249 | 6,639 | 4,078 | 1,353 | 26,716 |
| Jan | 4 | Bottom | 0,208 | 4,304 | 2,876 | 1,980 | 20,656 |
| Jan | 5 | Surface | 0,277 | 2,033 | 2,088 | 0,869 | 7,348 |
| Jan | 5 | Bottom | 0,297 | 2,130 | 3,980 | 1,006 | 7,170 |
| Jan | 6 | Surface | 0,348 | 6,744 | 3,436 | 1,172 | 19,396 |
| Jan | 6 | Bottom | 0,289 | 5,250 | 2,509 | 0,999 | 18,170 |
| Jan | 7 | Surface | 0,264 | 2,316 | 3,945 | 1,075 | 8,787 |
| Jan | 7 | Bottom | 0,535 | 2,374 | 3,169 | 0,838 | 4,434 |
| Jan | 8 | Surface | 0,325 | 8,203 | 3,670 | 1,917 | 25,219 |
| Jan | 9 | Surface | 0,630 | 9,788 | 3,891 | 1,544 | 15,527 |
| Jan | 9 | Bottom | 0,515 | 7,803 | 3,403 | 1,699 | 15,147 |
| **Month** | **Station** | **Layer** | **P-PO4 (μmol/l)** | **N-NO3 (μmol/l)** | **Si-SiO4 (μmol/l)** | **N-NH3 (μmol/l)** | **N-NO4/P-PO4** |
| May | 1 | Surface |  | 9,086 | 9,219 | 2,716 | 98,641 |
| May | 1 | Bottom | 0,046 | 4,821 | 7,490 | 4,318 | 103,693 |
| May | 2 | Surface | 0,236 | 20,003 | 24,545 | 8,045 | 84,691 |
| May | 2 | Bottom | 0,076 | 7,496 | 9,062 | 5,640 | 99,078 |
| May | 3 | Surface | 0,256 | 9,654 | 12,020 | 3,195 | 37,681 |
| May | 3 | Bottom | 0,250 | 10,049 | 11,235 | 9,626 | 40,241 |
| May | 4 | Surface | 0,305 | 8,990 | 9,817 | 2,584 | 29,482 |
| May | 4 | Bottom | 0,158 | 6,466 | 6,474 | 1,665 | 40,961 |
| May | 5 | Surface | 0,174 | 7,275 | 6,474 | 1,208 | 41,736 |
| May | 5 | Bottom | 0,194 | 7,380 | 6,530 | 1,051 | 37,985 |
| May | 6 | Surface | 0,406 | 23,305 | 19,878 | 10,360 | 57,447 |
| May | 6 | Bottom | 0,451 | 24,190 | 21,748 | 12,828 | 53,607 |
| May | 7 | Surface | 0,202 | 7,413 | 6,821 | 0,155 | 36,699 |
| May | 8 | Surface | 0,176 | 7,200 | 6,676 | 0,798 | 40,941 |
| May | 9 | Surface | 0,151 | 5,979 | 5,666 | 0,708 | 39,662 |
| May | 9 | Bottom | 0,150 | 5,259 | 6,478 | 1,105 | 34,965 |
| May | 10 | Surface | 0,179 | 8,061 | 8,394 | 0,759 | 44,987 |
| May | 10 | Bottom | 0,295 | 12,831 | 14,078 | 1,895 | 43,471 |
| May | 11 | Surface | 0,129 | 5,875 | 6,851 | 4,131 | 45,700 |
| May | 11 | Bottom | 0,158 | 6,250 | 6,995 | 4,897 | 39,571 |
