# Supplementary material for: Phytoplankton dynamics in a shellfish farming lagoon in a deltaic system threatened by ongoing climate change
Source: Sci Rep. 2024 Aug 21;14:19424. doi: 10.1038/s41598-024-70492-6 (PMC11339385; doi:10.1038/s41598-024-70492-6)
Supplement: Supplementary file 8 — Supplementary Table 2. [file 41598_2024_70492_MOESM8_ESM.docx]

**Table S2. Mesocosm experiment:** a) total phytoplankton biomass (Chl a μg/l) and b) percentage contribution of size classes micro- nano- and picophytoplankton within the chamber (IN) and outside the chamber (OUR) for each mesocosm (I, II and III).

**a)**

**IN**

|  | **I** | | | **II** | | | **III** | | |
| --- | --- | --- | --- | --- | --- | --- | --- | --- | --- |
| ***T0*** | 14,78 | 13,74 | 15,36 | 10,02 | 11,13 | 10,52 | 9,17 | 9,87 | 9,56 |
| ***T6*** | 16,07 | 16,32 | 16,64 | 21,88 | 20,12 | 20,75 | 17,17 | 17,46 | 16,13 |
| ***T12*** | 13,41 | 12,76 | 13,27 | 4,16 | 8,54 | 8,94 | 12,48 | 11,87 | 12,84 |

**OUT**

|  | **I** | | | **II** | | | **III** | | |
| --- | --- | --- | --- | --- | --- | --- | --- | --- | --- |
| ***T0*** | 69,23 | 72,63 | 72,19 | 67,43 | 67,92 | 67,91 | 68,73 | 68,25 | 68,13 |
| ***T6*** | 68,33 | 69,19 | 72,19 | 65,64 | 67,84 | 67,63 | 66,36 | 67,37 | 68,64 |
| ***T12*** | 57,98 | 55,64 | 56,27 | 53,86 | 54,22 | 55,38 | 48,94 | 50,18 | 49,73 |

**b)**

**IN**

|  | **Microfraction (%)** | | | | | | | | |
| --- | --- | --- | --- | --- | --- | --- | --- | --- | --- |
|  | **I** | | | **II** | | | **III** | | |
| ***T0*** | 14,78 | 13,74 | 15,36 | 10,02 | 11,13 | 10,52 | 9,17 | 9,87 | 9,56 |
| ***T6*** | 13,41 | 12,76 | 13,27 | 4,16 | 8,54 | 8,94 | 12,48 | 11,87 | 12,84 |
| ***T12*** | 10,64 | 8,35 | 9,23 | 1,74 | 2,12 | 1,94 | 5,12 | 6,24 | 7,03 |
|  | **Nanofraction (%)** | | | | | | | | |
| ***T0*** | 69,23 | 72,63 | 72,19 | 67,43 | 67,92 | 67,91 | 68,73 | 68,25 | 68,13 |
| ***T6*** | 57,98 | 55,64 | 56,27 | 53,86 | 54,22 | 55,38 | 48,94 | 50,18 | 49,73 |
| ***T12*** | 33,66 | 31,39 | 32,98 | 35,79 | 31,40 | 33,28 | 29,52 | 30,19 | 31,97 |
|  | **Picofraction (%)** | | | | | | | | |
| ***T0*** | 15,99 | 13,63 | 12,44 | 22,56 | 20,96 | 21,57 | 22,11 | 21,88 | 22,31 |
| ***T6*** | 28,61 | 31,60 | 30,45 | 41,98 | 37,24 | 35,69 | 38,58 | 37,95 | 37,44 |
| ***T12*** | 55,69 | 60,27 | 57,79 | 62,48 | 66,48 | 64,79 | 65,36 | 63,58 | 61,00 |

**OUT**

|  | **Microfraction (%)** | | | | | | | | |
| --- | --- | --- | --- | --- | --- | --- | --- | --- | --- |
|  | **I** | | | **II** | | | **III** | | |
| ***T0*** | 16,07 | 16,32 | 16,64 | 21,88 | 20,12 | 20,75 | 17,17 | 17,46 | 16,13 |
| ***T6*** | 17,95 | 16,25 | 16,28 | 20,45 | 20,28 | 19,28 | 27,25 | 25,37 | 24,39 |
| ***T12*** | 12,53 | 13,28 | 11,28 | 11,25 | 10,29 | 12,19 | 9,74 | 11,84 | 10,38 |
|  | **Nanofraction (%)** | | | | | | | | |
| ***T0*** | 68,33 | 69,19 | 72,19 | 65,64 | 67,84 | 67,63 | 66,36 | 67,37 | 68,64 |
| ***T6*** | 68,33 | 67,37 | 65,39 | 60,14 | 62,39 | 60,30 | 53,16 | 57,39 | 55,40 |
| ***T12*** | 63,06 | 61,74 | 64,39 | 66,15 | 64,29 | 64,39 | 61,72 | 59,81 | 60,73 |
|  | **Picofraction (%)** | | | | | | | | |
| ***T0*** | 15,60 | 14,49 | 11,17 | 12,48 | 12,04 | 11,62 | 16,46 | 15,18 | 15,24 |
| ***T6*** | 13,71 | 16,38 | 18,34 | 19,42 | 17,34 | 20,43 | 19,59 | 17,25 | 20,22 |
| ***T12*** | 24,41 | 24,99 | 24,34 | 22,61 | 25,43 | 23,42 | 28,54 | 28,35 | 28,90 |
