# Supplementary material for: Phytoplankton dynamics in a shellfish farming lagoon in a deltaic system threatened by ongoing climate change
Source: Sci Rep. 2024 Aug 21;14:19424. doi: 10.1038/s41598-024-70492-6 (PMC11339385; doi:10.1038/s41598-024-70492-6)
Supplement: Supplementary file 10 — Supplementary Table 4. [file 41598_2024_70492_MOESM10_ESM.docx]

**Table S4.** Blue crab (*Callinectes sapidus*) caught (kg) in the Po Delta between 2022 and 2024.

| **Years** | **Jan** | **Feb** | **Mar** | **Apr** | **May** | **Jun** | **Jul** | **Aug** | **Sept** | **Oct** | **Nov** | **Dec** | **TOTAL** |
| --- | --- | --- | --- | --- | --- | --- | --- | --- | --- | --- | --- | --- | --- |
| **2022** | 16 | 178 | 1.580 | 2.380 | 3.598 | 6.462 | 3.907 | 7.636 | 9.143 | 14.154 | 11.625 | 8.387 | **69.065** |
| **2023** | 7.051 | 2.767 | 19.446 | 18.490 | 14.385 | 13.066 | 41.316 | 291.538 | 148.674 | 90.763 | 132.134 | 83.648 | **863.276** |
| **2024** | 39.812 | 65.116 | 113.629 | 126.039 | 57.115 |  |  |  |  |  |  |  | **401.711** |
| ***TOT*** | ***46.879*** | ***68.061*** | ***134.655*** | ***146.909*** | ***75.097*** | ***19.528*** | ***45.223*** | ***299.173*** | ***157.817*** | ***104.917*** | ***143.759*** | ***92.035*** | ***1.334.052*** |
